# Applications of Point-of-Care-Ultrasound in Neonatology: A Systematic Review of the Literature

**DOI:** 10.3390/life14060658

**Published:** 2024-05-22

**Authors:** Florian Recker, Florian Kipfmueller, Agnes Wittek, Brigitte Strizek, Lone Winter

**Affiliations:** 1 Department of Obstetrics and Gynecology, University Hospital Bonn, Venusberg Campus 1, 53127 Bonn, Germany; agnes.wittek@ukbonn.de (A.W.); brigitte.strizek@ukbonn.de (B.S.); s4lowint@uni-bonn.de (L.W.); 2 Department of Neonatology and Pediatric Intensive Care, Children’s Hospital University of Bonn, Venusberg Campus 1, 53127 Bonn, Germany; florian.kipfmueller@ukbonn.de

**Keywords:** point-of-care-ultrasound, POCUS, neonatology, newborns, applications

## Abstract

Point-of-care ultrasound (POCUS) integration into neonatology offers transformative potential for diagnostics and treatment, enhancing immediacy and precision of clinical decision-making in this vulnerable patient population. This systematic review aims to synthesize evidence on POCUS applications, benefits, challenges, and educational strategies in neonatology. Literature search was conducted using SPIDER scheme keywords and MeSH terms related to POCUS and neonatology. Studies focusing on POCUS applications, its impact on clinical outcomes, and educational interventions for skill acquisition were included and analyzed using standardized tools, followed by a narrative synthesis of the findings. The search yielded 68 relevant publications, encompassing original research, reviews, and guidelines. POCUS applications varied across cardiovascular, pulmonary, neurological, and abdominal assessments. Key benefits included a reduced need for invasive procedures and rapid bedside diagnosis. Challenges included steep learning curves for clinicians and the need for standardized training and guidelines. Educational strategies highlighted the effectiveness of simulation-based training in enhancing ultrasound proficiency among neonatal care providers. POCUS represents a significant advancement in neonatal medicine, offering benefits for patient care. Addressing identified challenges through comprehensive training programs and developing standardized guidelines is crucial for optimized use. Future research should focus on evaluating educational outcomes and long-term impacts of POCUS integration into neonatal care.

## 1. Introduction

The integration of point-of-care ultrasound (POCUS) into neonatal medicine represents a pivotal shift towards more dynamic, real-time diagnostic and therapeutic capabilities [1]. This advanced imaging modality, characterized by its portability, versatility, and non-invasiveness, has rapidly emerged as an invaluable tool in neonatal intensive care units (NICUs) and beyond. POCUS extends the diagnostic acumen of clinicians by providing immediate visual insights into the physiological and pathological states of neonates, thereby facilitating prompt and informed clinical decision-making. The advent of POCUS in neonatology underscores a broader trend in medicine towards enhancing patient care through technological innovation [2].

Neonatology, a field inherently focused on a population at the extremities of human viability, presents unique diagnostic and therapeutic challenges. Neonates, particularly those who are premature or critically ill, require meticulous and often urgent medical interventions. Traditional imaging modalities, while informative, are sometimes limited by logistical constraints, the need for transport, and concerns over radiation exposure. POCUS, by contrast, offers a compelling alternative. It enables bedside assessment of intensive care patients, including preterm and critically ill neonates, as transporting these patients poses a risk of instability, and bypasses many of the limitations associated with conventional imaging techniques. Its applications span across multiple domains including, but not limited to, cardiovascular, pulmonary, neurological, gastrointestinal, and renal systems. For instance, cardiac POCUS can rapidly assess structural and functional heart anomalies critical in managing congenital heart diseases or hemodynamic instabilities. Similarly, lung POCUS can be instrumental in diagnosing pulmonary conditions, such as pneumothorax or pulmonary edema, without delay, ensuring that appropriate interventions can be initiated promptly. Bedside ultrasound has been integral to neonatology for decades, notably for tasks such as brain ultrasound to detect hemorrhages [3,4] and the ultrasound-based assessment of the patent ductus arteriosus [5], both of which are essential components of neonatologists’ POCUS repertoire. However, POCUS training remains suboptimally implemented in numerous countries worldwide. In resource-limited regions where access to other imaging modalities is scarce, the use of pocket-sized portable POCUS devices is not widespread, despite their advantages in terms of functionality and cost-effectiveness [6].

Despite the clear advantages of POCUS in enhancing neonatal care, its integration into clinical practice is not without challenges [7]. Foremost among these is the steep learning curve associated with acquiring the requisite skills for proficient use of ultrasound technology. The precise acquisition and interpretation of ultrasound images demand a deep understanding of neonatal anatomy and pathophysiology, coupled with technical ultrasound skills [8]. This necessitates comprehensive and ongoing training programs tailored specifically for neonatologists and NICU staff. Moreover, the field of neonatal POCUS is evolving, with ongoing debates and research regarding its optimal applications, protocols, and guidelines. Establishing standardized training curricula, certification processes, and clinical guidelines are crucial steps towards ensuring consistent and effective use of POCUS across different clinical settings.

The literature on POCUS in neonatology, while growing, presents a fragmented view of its applications, benefits, and limitations. This systematic literature review aims to highlight the transformative potential of POCUS in neonatal care by synthesizing evidence from the field of neonatology. It endeavors to inform clinical practice, shape educational strategies, and catalyze the formulation of consensus guidelines. Ultimately, the review aims to advance the understanding and utilization of POCUS in neonatology, optimizing care for one of the most vulnerable patient populations and paving the way for improved clinical outcomes.

## 2. Materials and Methods

This systematic search of the literature was conducted following the updated Preferred Reporting Items for Systematic Reviews and Meta-Analyses (PRISMA) statement on reporting systematic reviews and meta-analyses of studies [9,10]. The literature search was conducted in PubMed and Google Scholar, using a combination of keywords and MeSH terms, such as “point-of-care ultrasound”, “neonatology”, “ultrasound education”, “peer-assisted learning”, and “clinical skills training”. These terms were combined using Boolean operators to capture a broad spectrum of the relevant literature. In the study selection process, no restrictions were made based on the quality of studies, the study design, and the publication date. Studies published in German or English up to 22 February 2024, that investigated POCUS applications in neonatal care were included in the data extraction process. These inclusion criteria are designed to offer detailed and valuable insights into diverse POCUS applications in neonatal care. To augment the electronic search, the reference lists of identified studies and pertinent reviews were manually scrutinized for additional sources. Efforts were also be made to identify grey literature to ensure comprehensiveness.

A systematic and structured data extraction process was implemented. Independent reviewers (L.W., F.R., and F.K.) conducted initial screenings based on titles and abstracts, proceeding to full-text reviews for selected articles. A blinded approach was used. Any differences among the authors concerning inclusion were resolved through direct discussion. After the exclusion of articles due to duplication, non-fulfillment of inclusion criteria, inaccessibility of full-text versions, and languages other than German or English, a standardized data extraction was conducted. 

Data from the included studies underwent a meticulous narrative synthesis process, providing a qualitative overview of the field. In original studies, data on the targeted study characteristics and designs, participants’ demographics, types of applicated POCUS, and POCUS findings in various indications and applications were collected, according to the SPIDER (Sample, Phenomenon of Interest, Design, Evaluation, Research Type) scheme (see Table 1 and Supplementary Table S1) [11]. Data concerning POCUS applications and findings, POCUS training specifics, including curriculum details and instructional methods, and outcomes related to knowledge and skill acquisition were extracted from included reviews and guidelines (see Supplementary Tables S2 and S3).

The methodological quality of the included studies was rigorously assessed using the Medical Education Research Study Quality Instrument (MERSQI). This evaluation focused on the study design, methodology, sample size, reliability and validity of assessment tools, statistical analysis methods, and the relevance of outcomes to educational objectives. This quality assessment helped identify the strength of evidence and potential biases in the included studies.

## 3. Results

### 3.1. Search Results

**Figure 1:**
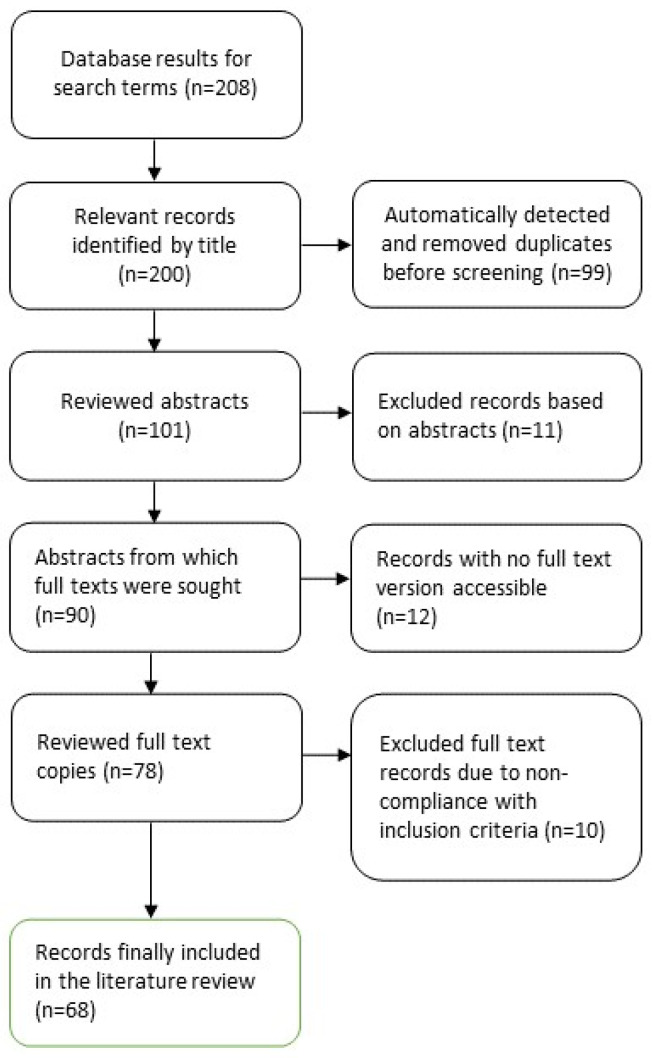
Literature selection process according to the Preferred Reporting Items for Systematic Reviews and Meta-Analyses (PRISMA) statement [9,10].

The literature search returned 208 records, of which 200 were considered relevant based on their titles. Following the exclusion of duplicates (n = 99), records with unsuitable abstract content (n = 11), publications lacking accessible full-text versions (n = 12), and those not meeting the inclusion criteria upon full-text review (n = 10), the search yielded a total of 68 publications dedicated to the applications of POCUS in neonatology (refer to Figure 1). In total, 33 original studies, comprising prospective interventional and observative studies, retrospective studies, cross-sectional studies, and case reports were included and examined according to the SPIDER scheme concerning potential scopes of POCUS in neonatology (see Supplementary Table S1). Furthermore, reviews of the literature (n = 30) (see Supplementary Table S2) as well as guidelines and proposed protocols (n = 5) (see Supplementary Table S3) for using POCUS in neonatology were included in this review.

### 3.2. Applications of POCUS in Neonatology

**Figure 2:**
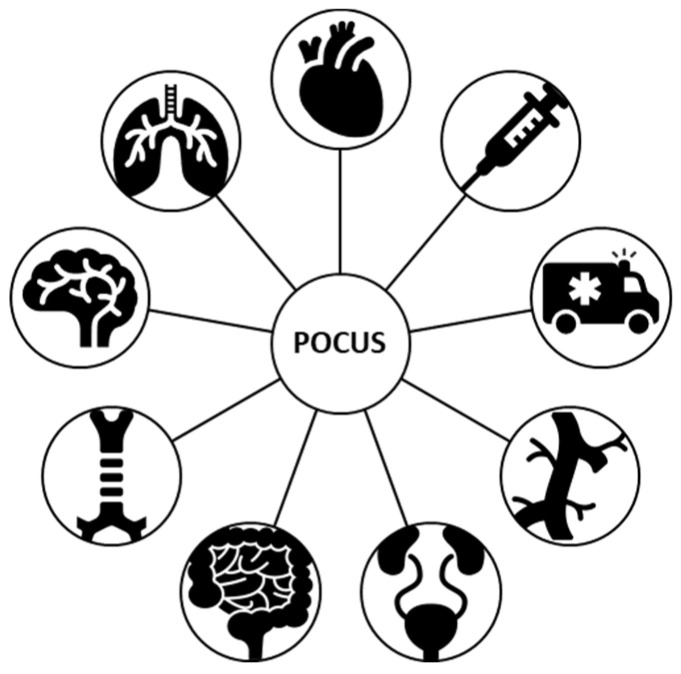
Multi-specialized applications of point-of-care ultrasound (POCUS) in neonatology.

Point-of-care ultrasound has revolutionized neonatal care by offering real-time diagnostic and procedural guidance at the bedside. In neonatology, POCUS finds extensive application across numerous organ systems, enabling clinicians to assess and manage critical conditions in premature and term newborns in a timely manner. The multi-specialization of POCUS utilizations is illustrated in Figure 2.

In general, POCUS applications can be categorized into diagnostic and procedural scopes (see Table 2), which are further described below.

#### 3.2.1. Diagnostic Applications 

**Figure 3:**
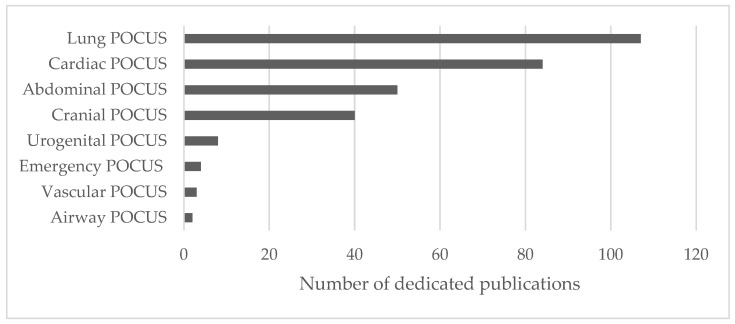
Quantitative analysis of POCUS applications in neonatology.

In the realm of diagnostics, POCUS serves as a versatile aid in assessing various aspects of neonatal physiology. A quantitative evaluation of the diagnostic applications of POCUS in neonatology revealed a considerable presence of pulmonary and cardiac POCUS followed by abdominal and cranial POCUS in the literature. Urogenital, emergency, vascular, and airway applications appeared to be less represented in included publications (see Figure 3).

##### Cardiac POCUS

Cardiac assessment represents the most prevalent area of POCUS utilization [26]. POCUS emerges as a pivotal tool, offering basic, time-sensitive evaluations aimed at addressing specific clinical questions, aiding in urgent or emergent decision-making, and guiding resuscitative interventions [16,23], which is particularly crucial in resource-limited settings where comprehensive echocardiography and pediatric cardiology consultations may be lacking [33].

Real-time assessment of global cardiac function, as well as right and left ventricular performance, provides insights into hemodynamic pathophysiology. This assessment encompasses various parameters, including contractility, cardiac filling status, cardiac output, and heart rate. It involves evaluating myocardial performance through both qualitative eyeballing and quantitative measurements, such as ejection fraction, myocardial strain, tricuspid annular plane systolic excursion (TAPSE), and mitral annular plane systolic excursion (MAPSE) [2,13,18,19]. Based on the assessment of the cardiac filling status in acute cardiovascular collapse, the corresponding indicated therapy can be applied. For example, the underfilled heart with reduced preload requires fluid therapy, whereas the overloaded heart with increased preload requires inotrope therapy [16].

Furthermore, POCUS facilitates the assessment of systemic blood flow dynamics by measuring blood flow velocity and cross-sectional areas over the aortic and pulmonary valves, with additional measurements of superior vena cava flow providing estimates of cerebral blood flow [19]. Assessment of the inferior vena cava, focusing on collapsibility and respiratory variation, provides valuable hemodynamic volume information [20,21].

In clinical scenarios involving persistent pulmonary hypertension, POCUS aids in estimating pulmonary artery systolic pressure, assessing shunt direction, and evaluating ventricular size, symmetry, septal position, and function, thereby elucidating information on diastolic and systolic pressures. Moreover, parameters such as the pulmonary arterial acceleration time (PAAT) to right ventricular ejection time (RVET) ratio offer valuable insights into pulmonary vascular resistance [2,18,19,78,79]. In this setting, POCUS assessment facilitates the differentiation between pre- and postcapillary pulmonary hypertension and the recognition of cardiopulmonary interactions, which enables physicians to tailor individualized pathophysiology-based treatment strategies [80,81]. 

In neonatal resuscitation, POCUS assists in distinguishing pseudo-pulseless electrical activity (pseudo-PEA) from true-PEA, aiding in appropriate intervention [29]. Furthermore, it aids in identifying reversible causes of cardiac arrest during episodes of shock and hemodynamic instability, ranging from pericardial effusions to ventricular dysfunction [14]. Pericardial effusion and cardiac tamponade are detectable through POCUS, guiding clinicians to discern their hemodynamic significance within the clinical context [23].

Additionally, POCUS facilitates the detection of congenital structural abnormalities such as tetralogy of Fallot, intracardiac shunts like patent ductus arteriosus, and valvular dysfunctions [18,19,23,82], along with aiding in the differentiation of normal fetal-to-neonatal circulation transitions from pathologic states [34]. 

Lastly, POCUS enables the visualization of intracardiac masses, offering diagnostic insights into various cardiac pathologies, including atrial thrombi, vegetations associated with endocarditis, and cardiac tumors [28].

Cardiac POCUS plays a crucial role in evaluating neonatal cardiac function, including myocardial performance, volume status, and systemic blood flow. It assists in identifying conditions such as persistent pulmonary hypertension, pericardial effusion, and cardiac tamponade. Additionally, it aids in detecting intracardiac shunts, valvular dysfunction, and congenital anomalies like tetralogy of Fallot. 

##### Lung POCUS

The application of lung POCUS in neonatology encompasses a wide range of indications and pathologies, each characterized by distinct ultrasound signs, patterns, and findings, facilitating rapid and accurate diagnosis at the bedside.

To distinguish a healthy from a diseased lung, an overview of normal lung patterns is given. POCUS reveals a hyperechoic pleural line exhibiting dynamic sliding with respiration, accompanied by horizontal reverberation artifacts, called A-lines, indicating the normal presence of air. The bat-wing sign, resulting from two adjacent rib shadows, and the sea-shore sign, indicating normal pleural sliding in motion (M)-mode, further delineate normal lung architecture and provide essential references for comparison [20,39,42].

Several lung pathologies, such as hyperinflation, atelectasis, air leak syndromes, edema, and consolidation, can be distinguished using POCUS [12,36]. In pulmonary edema, POCUS detects the presence of multiple B-lines, dense vertical lines emanating from the pleura into the lung parenchyma, indicating interstitial fluid accumulation. The sum of B-lines corresponds with the amount of edema [20]. Pulmonary consolidation represents areas of pulmonary hypoventilation, often marked by a liver-like appearance known as hepatization or tissue-like sign. In ultrasound imaging, alveolar collapse is identifiable by dynamic air bronchograms, presenting as undefined contours with hyperechoic images within the consolidation. Additionally, features of consolidated lung tissue include the shred sign, represented by a fragmented interface between the consolidated and aerated lung tissues; the barcode sign, indicating the absence of pleural sliding in M-mode; and the presence of the lung pulse, a phenomenon where cardiac motion is transmitted to the pleura through the consolidated lung tissue [20,35,39,42].

Ultrasound facilitates diagnosis, quantification, and monitoring of a pleural effusion, which presents as an anechoic area between the parietal and visceral pleura. The jellyfish sign, showing the atelectatic lung floating in the dense black effusion, and the sinusoid sign, observed when respiratory variation reduces the distance between the parietal and visceral pleura separated by a pleural effusion, are indicative features. In the sonographic image, the quad sign is further depicted, which is an irregular rectangle formed by two lateral rib shadows, the parietal pleura, and the visceral pleura or lung [18,20,35].

Pneumothorax is a common neonatal complication that particularly manifests in the setting of positive pressure ventilation. It is characterized by the stratosphere or barcode sign, indicating the absence of pleural sliding in M-mode, and the absence of lung pulse in the pneumothorax area. The lung point sign, which visualizes the point of separation of the visceral pleura from the chest wall parietal pleura, serves as a diagnostic feature. These distinctive signs aid clinicians in promptly recognizing and managing pneumothorax [2,18,20,35]. 

Additionally, specific diseases and conditions occurring in neonatology can be diagnosed using POCUS, allowing prompt treatment initiation. These include the transient tachypnoea of the newborn (TTN), characterized by the double lung point sign. This sign indicates a sonographic difference in lung echogenicity between the upper, normally aerated lung fields and the lower, edematous lung fields. Highly specific for TTN, with a reported specificity of up to 100%, it serves as a key diagnostic feature. Additionally, mild changes in the pleural line and mild pleural effusion may be present [35,40]. Neonatal acute respiratory distress syndrome, a major neonatal complication, presents in POCUS with homogeneous alveolar-interstitial edema, resulting in a bilateral “white lung” appearance, along with the presence of B-lines and absence of A-lines. Other features include an irregular or thickened pleural line, consolidations of variable size that are predominantly posterior, subclinical pleural effusion, and air bronchograms [18,21,35,39]. Meconium aspiration syndrome exhibits a stained pattern with areas of consolidations accompanied by air bronchograms, which are bilateral and of variable size. It may also present with an alveolar-interstitial pattern or a normal pattern, along with pleural effusion and dynamic features [18,21,35,38]. 

Inflammatory pulmonary diseases in newborns, diagnosable using POCUS, encompass pneumonia, bronchiolitis, and SARS-CoV-2 virus infection. Congenital pneumonia shows a nonspecific pattern that varies depending on severity, with areas of consolidations having irregular margins. Other features include an irregularly thickened pleural line, subclinical pleural effusion, and dynamic air bronchograms within consolidations [18,35]. Additionally, the severity of bronchiolitis can be assessed using POCUS. Moderate extent correlates with predominantly non-specific findings, such as patchy interstitial pattern marked by the presence of B-lines and a white lung pattern, along with subpleural consolidations. Furthermore, the severity of the interstitial ultrasound pattern is somewhat correlated with an increased need for respiratory support [42]. In SARS-CoV-2 virus infection, abnormalities to identify include B-lines; interstitial patterns; an irregular, interrupted, or thickened pleural line; and consolidations of different extents, predominantly posterior. POCUS is valuable for evaluating lung involvement in SARS-CoV-2 infections, correlating with disease severity and the need for respiratory support [43].

In assessments of lung development and congenital abnormalities, POCUS aids in predicting bronchopulmonary dysplasia, diagnosing conditions like congenital diaphragmatic hernia and chylothorax, and in monitoring the response to surfactant therapy in neonates [2,17,18,35]. Assessing ductus arteriosus flow, early postnatal ventricular disproportion, and pulmonary hypertension—indicated by an index of approximately 0.3, calculated from the pulmonary artery acceleration time to the right ventricular ejection time—represent feasible parameters for predicting outcomes in neonates with congenital diaphragmatic hernia [78,79,82]. Furthermore, diaphragmatic motion assessment using M-mode allows for quantitative evaluation of diaphragmatic excursion, with 0.5 to 1 cm serving as an acceptable range, providing valuable insights into respiratory function.

During neonatal resuscitation, lung POCUS quantifies lung aeration, monitors alveolar recruitment, and guides ventilation maneuvers, ensuring optimal respiratory support and enhancing neonatal outcomes [14,35].

##### Cranial POCUS

Cranial POCUS enables the non-invasive assessment of intracranial structures by visualizing a neonate’s brain and ventricular spaces through the anterior fontanelle and the mastoid window. The anterior fontanelle, patent in over 75% of infants at 12 months, serves as the required acoustic window. Additionally, the mastoid window facilitates the visualization of infratentorial structures, including the cerebellum [14,44]. 

POCUS aids in detecting intraventricular, parenchymal, extradural, and subdural hemorrhages [22,36,44] as well as periventricular leukomalacia [26] and hydrocephalus [12]. Hydrocephalus, for instance, can be assessed using cranial POCUS by examining ventricular sizes. In the coronal plane, a widening of the anterior horn of the lateral ventricles beyond 10 mm results in a ‘Mickey Mouse ears’ appearance, while an enlargement of the temporal horn surpassing 3 mm is notable in hydrocephalus. In the sagittal plane, an enlargement of the third ventricle exceeding 1 mm is indicative. An important indicator of hydrocephalus is a bifrontal index exceeding 0.5 [22,45,46]. 

Additionally, POCUS can aid in identifying true positive skull fractures by visualizing cortical irregularities from multiple orientations [47]. Other conditions such as cerebral midline shift [22], cranial shunt failure [48], cerebral sinovenous thrombosis [49], and hypoxic-ischemic encephalopathy [18,24] can also be diagnosed and monitored using POCUS. Moreover, transcranial Doppler ultrasound allows for the assessment of cerebral blood flow by visualizing and examining the flow velocity and resistance of cerebral arteries [14]. 

Ocular ultrasound serves multiple purposes, including assessing ocular movement post-trauma, ensuring retinal integrity, and identifying the optic nerve [14] to conduct measurements of the optic nerve sheath diameter, providing insights into intracranial pressure levels [48].

##### Airway POCUS

Airway POCUS involves assessing the trachea, which appears as an anechoic round structure surrounded by hyperechoic cartilage rings. Additionally, it allows for the evaluation of vocal cord mobility and detection of subglottic airway issues [12]. 

##### Abdominal POCUS

Abdominal POCUS serves as a valuable tool for detecting various abnormalities within the abdominal cavity. 

This includes identifying major organ abnormalities, such as hepatic hemangiomas [15,24]. Furthermore, it allows for the detection of intra-abdominal and pelvic free fluid, which may indicate conditions such as ascites or bowel/gut perforation [15,31,36]. Abdominal POCUS also aids in assessing mesenteric blood flow and detecting signs of ischemia [22]. Moreover, it is instrumental in diagnosing neonatal ileus, which is characterized by bowel distension, reduced peristalsis, thinning of bowel wall thickness to less than 1 mm, and loss of normal layering of the bowel. This results in the hyperechogenicity of mucosa and muscularis propria, along with thickened parallel valvulae conniventes, which manifests as a distinctive ‘zebra’ pattern [51].

Furthermore, necrotizing enterocolitis, a severe complication in newborns, can be identified using abdominal POCUS based on various sonographic features. These include pneumatosis intestinalis, characterized by a hyperechoic granular pattern of the bowel wall with posterior reverberation artifacts that remain unchanged with peristalsis. Additionally, portal venous gas appears as intravenous echogenic foci moving with the blood flow, while pneumoperitoneum is identified based on the peritoneal stripe sign. Loss of normal bowel wall layering, free peritoneal fluid, thickening, and secondary thinning of the bowel wall, hyperemia, and a secondary decrease in vascularization due to necrosis can be observed with a note of caution regarding perforation [2,21,22,36,50,51].

Meconium peritonitis can result from in utero bowel perforation and presents antenatally with fetal ascites, dilated intestinal loops, intraabdominal calcifications, echogenic bowel, polyhydramnios, and pseudocysts. Postnatally, ultrasound reveals a dilated proximal to inspissated meconium bowel, distal dilated bowel, homogenous and anechoic ascites with internal septa, a snowstorm appearance in perforation cases, meconium pseudocysts, and pneumoperitoneum [51].

POCUS can also be used to diagnose intestinal positioning anomalies. In cases of incarcerated inguinal hernia, abdominal POCUS shows a segment of the intestine presenting with wall thickening and hyperechogenicity. Color Doppler and microvascular US aid in differentiating incarcerated and strangulated hernias by identifying the absence of internal vascularity [51]. Intussusception can be visualized in a transverse view, revealing a target or doughnut-like mass, and in a longitudinal view, showing the pseudokidney sign [52]. Malrotation with a midgut volvulus can be assessed using abdominal POCUS by examining the relationship between the small mesenteric artery and vein, identifying distal duodenal obstruction and proximal duodenal dilatation, non-retroperitoneal position of the third portion of the duodenum, and the whirlpool sign [51]. In contrast, the segmental intestinal volvulus is unrelated to malrotation and shows a normal orientation of the small mesenteric arteria and vein, a normal retroperitoneal course of the third portion of the duodenum, and a normal midgut rotation [51]. 

Further abdominal POCUS applications include aiding in gastric evaluation, such as determining gastric emptying, content, and volume, which is crucial for assessing the risk of pulmonary aspiration during sedation. Gastric volumes equal to or less than 1.25 mL/kg in children are considered a low aspiration risk [21,53,54]. Abdominal POCUS also serves as a guide for enteral nutrition in intensive care unit patients, allowing for the management and monitoring of feeding, prediction of feeding intolerance by ultrasound meal accommodation test, and assessment of treatment response by examining gastrointestinal dynamics like gastrointestinal diameter, mucosal thickness, peristalsis, gastric residual volume, and blood flow [53]. Furthermore, it facilitates investigations of gastric foreign bodies, assists in diagnosing conditions like hypertrophic pyloric stenosis, and is essential for monitoring gastric insufflation during mechanical ventilatory support [54]. 

##### Urogenital POCUS

Urogenital POCUS is instrumental in various diagnostic and monitoring tasks. It enables bladder volume measurement, facilitating the assessment of urinary retention, as well as aiding in estimating oliguria and anuria [14,36]. Additionally, it plays a crucial role in detecting obstructive uropathy [24]. 

##### Vascular POCUS

Vascular POCUS allows for the detection of deep vein thrombosis, enabling timely intervention to prevent complications [24]. Furthermore, it aids in identifying vena cava thrombosis [17] and acute critical aortic occlusion, which can arise from coarctation or thrombosis [25], ensuring prompt management. 

##### Emergency POCUS

Emergency POCUS in neonatology plays a pivotal role in critical care scenarios, particularly when standard diagnostics are not conclusive, standard interventions are not successful, or if a rapid diagnosis is imperative. Indications for emergency POCUS include acute circulatory shock unresponsive to the Neonatal Resuscitation Program protocol without an identifiable cause, acute respiratory distress syndrome along with worsening hypoxemia not alleviated by typical respiratory support, and unexplained drops in hemoglobin exceeding 20% within 24 h, raising suspicion of acute bleeding [2]. 

Emergency POCUS protocols provide a rapid assessment of critical conditions in these scenarios. The Focused Cardiac Ultrasound (FOCUS) protocol aims to assess cardiac function and volume status by evaluating hemodynamic function, identifying the presence or absence of pericardial effusion, assessing cardiac chamber size, and estimating systolic pressure [14]. Another crucial protocol is the Focused Assessment with Sonography in Trauma (FAST) protocol, used for the rapid detection of hemoperitoneum, hemopericardium, hemothorax, and pneumothorax, enabling timely intervention to stabilize the patient and mitigate further injury [14,21]. Additionally, the Rapid Assessment of the Neonate with Sonography (RANS) protocol is specifically designed for neonatal emergencies. This protocol aids in the prompt diagnosis of decompensating neonates by identifying critical conditions such as pericardial effusion, pneumothorax, pleural effusion, central venous line malposition, and severe intraventricular hemorrhage [21]. 

Overall, emergency POCUS protocols provide rapid and non-invasive identification of critical conditions, guiding critical interventions and optimizing patient care in emergency settings.

#### 3.2.2. Procedural Applications

Procedural POCUS applications in neonatology encompass a wide range of essential clinical procedures, aiding in rapid and accurate diagnoses and interventions. These applications are vital in neonatal intensive care units and emergency settings, ensuring timely and effective management of critically ill neonates.

One fundamental aspect of procedural POCUS is the assessment of neonatal endotracheal tube (ETT) placement, sizing, and depth confirmation, providing real-time guidance and assessment and minimizing the risk of complications during intubation procedures [21,56]. This is a common procedure in both neonatal intensive care units facing respiratory failure and emergency settings like neonatal resuscitation [58]. Firstly, POCUS assists in verifying ETT positioning to rule out potential complications, such as esophageal or endobronchial intubation. In esophageal intubation settings, characteristic ultrasound findings include a hyperechoic circular structure adjacent to the trachea, often accompanied by a hyperechoic shadow within the esophageal lumen, known as the double trachea sign. In contrast, endotracheal intubation is identified by the presence of a hyperechoic shadow within the hyperechoic cartilage of the trachea, creating parallel hyperechoic arcs, referred to as the railroad sign. Additionally, scanning the bilateral anterior lungs for pleural sliding aids in distinguishing between endobronchial and endotracheal intubation, with unilateral pleural sliding indicating the former and bilateral pleural sliding indicating the latter [21,59]. Moreover, POCUS facilitates the determination of the optimal ETT depth from the gum line, which is crucial for preventing complications such as mainstem bronchus intubation or dislodgement. A simple formula by Halm et al. ensures proper positioning within the trachea: Optimal ETT depth from gum (cm) = 5.21 + 1.03 × weight (kg) [45]. Furthermore, ETT sizing can be guided using POCUS to minimize laryngeal trauma and swelling. By measuring the transverse air column diameter at the level of the cricoid cartilage, clinicians can estimate the maximum size for the outer diameter of the ETT, ensuring a precise fit [21]. 

Furthermore, POCUS aids in intubation settings by assessing vocal fold function, predicting post-extubation stridor, and guiding difficult laryngoscopy or cricothyrotomy. 

Procedural guidance for vascular access represents a further major application of the neonatal POCUS. Ensuring adequate catheter size and evaluating and precisely adjusting tip position in real-time reduces the risk of complications in the placement of peripherally inserted central catheters, umbilical arterial and venous central catheters, and peripheral arterial and venous lines. Optimal positioning of the catheter tip involves visualizing the line within the superior or inferior vena cava, proximal to the cavoatrial junction but not entering the right atrium [61,63,66,67,71,72]. Incorrect placement can lead to severe consequences, such as cardiac arrhythmias, perforation, pericardial effusion, and cardiac tamponade if the tip is inserted too deeply. Conversely, insertion too shallowly in noncentral veins can result in decreased hemodilution, extravasations, seizures or paraplegia, thrombosis, and even death [27,64]. Utilizing B-mode views, including the apical 4-chamber view, left parasternal short axis view, bicaval subcostal view, and high right parasternal longitudinal view, enables accurate visualization and tracking of the catheter tip until it reaches the target zone [63,68]. The study by Cowan et al. demonstrated the safety and efficacy of identifying central catheter tip location using POCUS in neonates, with no adverse effects on cardiorespiratory stability observed before and after the procedure [62]. The umbilical venous catheter stands out as one of the most commonly used central lines in neonatal care. This catheter provides stable intravenous access for infants requiring advanced resuscitation in the delivery room, cardiac catheterization, or administration of medications, fluids, and parenteral nutrition during the first days of life. Its reliability and ease of access make it an indispensable tool in neonatal intensive care, ensuring timely and effective treatment for critically ill neonates [14,68]. 

For the management of newborns undergoing extracorporeal membrane oxygenation, POCUS aids in evaluating candidacy, guiding cannulation procedures, ensuring correct catheter placement, and detecting and managing complications, including pneumothorax and effusions, by daily monitoring of heart and lung function as well as brain perfusion [14,75,82,83]. 

Other procedural applications of POCUS in neonatology include lumbar puncture guidance and regional and neuraxial blocks. Lumbar puncture, guided by ultrasound, enhances the precision of needle placement by identifying spinal landmarks and visualizing needle entry into the subarachnoid space, thereby reducing traumatic taps and improving success rates. Furthermore, POCUS can aid in ensuring the accurate placement of nasogastric and orogastric tubes within the body or the antrum of the stomach by visualizing the gastric tube as a hyperechoic line [21,59]. Fluid drainage procedures such as pericardiocentesis, thoracocentesis, and paracentesis are facilitated through POCUS real-time visualization and needle guidance, enhancing accuracy and reducing the risk of iatrogenic complications [2,59]. Suprapubic bladder aspiration [59], urinary catheter visualization [8], and abscess drainage [24] also benefit from POCUS guidance. 

POCUS serves as a versatile tool in neonatology, offering comprehensive diagnostic capabilities and procedural guidance, thereby enhancing clinical decision-making and improving outcomes for newborns in various clinical settings.

### 3.3. POCUS Guidelines in Neonatology

POCUS guidelines in neonatology offer structured approaches to using ultrasound in various clinical scenarios to aid in rapid assessment and decision-making. Several protocols have been developed by researchers and experts in the field to address specific needs in neonatal care (see Supplementary Table S3). 

The Crashing Neonate Protocol (CNP) introduced by Elsayed et al. is tailored for neonatal emergencies characterized by significant cardiorespiratory instability. It applies to both term and preterm infants. CNP serves as an adjunct to current neonatal resuscitation guidelines and involves a stepwise systematic targeted assessment using basic ultrasound views. These views include lung POCUS for pulmonary emergencies like pneumothorax or pleural effusion, cardiac POCUS for assessing shock and hemodynamic instability, cranial POCUS for acute brain hemorrhage, abdominal POCUS for evaluating abdominal emergencies, and central line POCUS for assessing complications related to central lines [31]. Also developed for the emergency management of newborns is the SAFE-R protocol reported by Yousef et al. It is designed for rapidly screening common life-threatening complications in suddenly decompensating infants in the neonatal intensive care unit. It includes, similarly to the CNP developed by Elsayed et al., cardiac, lung, abdominal, and cranial POCUS to assess for myocardial dysfunction, tension pneumothorax, acute abdominal complications, and intraventricular hemorrhage [25]. Furthermore, Hardwick and Griksaitis proposed an ultrasound examination approach to pediatric shock, involving a predefined sequence of focused scans including lung, cardiac, inferior vena cava, abdominal, and cranial POCUS [15]. 

Maddaloni et al. developed a congenital diaphragmatic hernia POCUS protocol that includes assessments of cardiac function, lung volumes, abdominal structures, and cerebral blood flow. This protocol is implemented at standardized timelines following postnatal stabilization, leading up to surgery, and after surgical correction [17]. 

The international evidence-based guidelines on POCUS for critically ill neonates and children issued by the POCUS Working Group of the European Society of Paediatric and Neonatal Intensive Care provide comprehensive recommendations for cardiac, lung, vascular, cerebral, and abdominal POCUS based on a thorough literature review. These guidelines offer valuable insights into the appropriate use of POCUS in various clinical contexts in neonatology [24]. 

### 3.4. Neonatology Considerations

When considering neonatology, it is crucial to acknowledge the unique anatomical and physiological features of newborns that significantly impact medical procedures and diagnostic imaging using POCUS. When performing airway POCUS, clinicians must recognize that neonates contain increased water content along with decreased adipose tissue and muscle mass compared to adults. Additionally, the lack of calcification in cartilaginous structures and the overall smaller size and more superficial anatomy of neonates contribute to better visualization of laryngeal structures compared to adults [21]. Similarly, in lung POCUS, the anatomical differences in pediatric patients allow for higher-quality images, particularly when visualizing deeper pulmonary parenchyma [21]. These differences enhance the diagnostic capabilities of ultrasound in assessing lung conditions in neonates. Thus, endotracheal intubation in neonates is challenging due to their narrow and short airways, especially in preterm newborns. Achieving precision in ETT placement requires a high level of skill and careful attention [58]. Furthermore, when performing line placements, such as central venous or arterial catheters, clinicians benefit from the ability to visualize deeper structures in pediatric patients. This facilitates safer and more accurate placement of lines, minimizing the risk of complications [21]. Moreover, cranial ultrasound in neonates represents an outstanding diagnostic modality and is commonly performed using the patent anterior fontanelle and mastoid window. These anatomical features provide accessible windows for ultrasound examination of the brain, enabling clinicians to assess for intracranial abnormalities with relative ease [14,44]. 

In summary, neonatology considerations encompass understanding and adapting to the unique anatomical and physiological characteristics of newborns. In particular, the optimized visualization achieved during procedures due to the increased ultrasound reach increases patient safety. Incorporating these considerations into medical procedures and diagnostic imaging techniques ensures safe and effective care for this vulnerable population.

### 3.5. Advantages and Disadvantages of POCUS in Neonatology

Utilizing POCUS in neonatology offers a multitude of advantages, revolutionizing the diagnostic and therapeutic landscape for newborns in critical care settings. However, some drawbacks, challenges, and burdens must also be considered when introducing the use of POCUS in neonatology.

#### 3.5.1. Advantages

POCUS facilitates the timely diagnosis of congenital or acquired abnormalities in newborns with complex clinical situations [49]. This enables timely, resuscitative, and lifesaving interventions and escalation of care prior to the development of more severe symptoms and complications, ultimately improving patient outcomes [2,14,50]. POCUS is non-invasive, lacks ionizing radiation, and provides quick, immediate feedback, allowing for ease of serial assessments and cost-effective imaging along with promoting time at the bedside of the critically unwell child [39,48]. Conducting repeated scans on the same patient enables healthcare providers to assess the impact of interventions effectively. In addition, patient safety during invasive procedures can be improved by real-time adjustments using POCUS [20,64]. The enhancement of diagnostic accuracy and imaging acquisition time using POCUS can lead to medical innovation and improve overall outcomes. POCUS has become an essential part of day-to-day practice in neonatology, similar to the stethoscope, and could be considered as an extension of the physical examination performed at the bedside by non-radiologists [36]. Because of distinct anatomical and physiological features, POCUS yields clear and detailed images relatively easily in the pediatric population [20], and it may complement or sometimes replace radiographic studies, reducing the ionizing radiation, the need for transport to fluoroscopy suites, and the costs [51,66]. 

#### 3.5.2. Disadvantages

The accuracy of POCUS is dependent on the skill of the physician performing the examination, and distinguishing between different types of fluids, such as blood versus serous fluid, can be challenging. While POCUS is widely used in adult medicine, the majority of the evidence base is in the adult population, posing a challenge for training in pediatric settings [20]. In terms of specific POCUS applications, for instance, the study by Halm et al. provided insufficient evidence to support the use of POCUS for identifying hydrocephalus [45]. Furthermore, cardiac POCUS may not be adequate for appraising and targeting the management of specific physiologic states, like pulmonary hypertension and patent ductus arteriosus, or the grading of cardiac dysfunction in neonates. For advanced conditions, targeted neonatal echocardiography or other modalities may be necessary [16,21]. 

## 4. Discussion

Many publications included in this review provided statements concerning the comparability of diagnostic accuracy and clinical utility between POCUS and traditional imaging modalities in neonatology. In the following, we will provide an overview of several statements on different POCUS applications. No quantitative analysis of superiority of any imaging modality is provided by this review.

In assessing central catheter tip location, the study by Amer et al. reports a POCUS sensitivity of 83% and specificity of 96% in relation to chest radiographs as a reference standard. Furthermore, the chest radiographs took about 4 seconds longer than POCUS [61]. The study by Zhang et al. confirms these findings. POCUS proves more accurate in positioning catheter tips and in detecting iatrogenic pericardial effusion promptly, resulting in a reduction of fatality rates and an improvement in the prognosis of infants with catheter-associated pericardial infusions [27]. The review by Pan et al. submits the statement that POCUS surpasses chest radiography in evaluating umbilical line tip location, demonstrating higher accuracy, quicker line placement, and reduced radiation exposure, underscoring its value in neonatal care [70]. 

For peripheral arterial line placement, POCUS seems to lead to fewer attempts for successful placement in infants above 2.5 kg compared to traditional landmark-based methods, indicating its efficacy in improving procedural outcomes [73]. 

The current gold standard for endotracheal tube verification is chest radiography [84,85]. Several studies, such as those by Ariff et al., Congedi et al., and Zaytseva et al., have demonstrated high sensitivity, ranging from 93.4% to 99.7%, and high agreement rates of 98.9% for POCUS compared to standard methods. POCUS exhibits substantial agreement with chest X-rays, providing a rapid and effective technique for confirming optimal ETT placement in neonates [56,58,60]. 

In diagnosing neonatal respiratory disorders, such as respiratory distress syndrome, pneumonia, meconium aspiration syndrome, pneumothorax, and pulmonary atelectasis, lung POCUS shows a significant agreement of 98.5% with chest radiography, proving to be a reliable bedside diagnostic tool with comparable safety and efficacy [38]. 

For sterile urine collection using suprapubic aspiration, in the study by Mahdipour et al., POCUS demonstrates the highest success rate (97.4%) compared to other methods such as traditional non-POCUS suprapubic aspiration and bladder catheterization [77]. This underscores its superiority in urine sampling.

The study by Parri et al. examined the accuracy of POCUS in identifying skull fractures. POCUS exhibits a sensitivity of 90.9% and a specificity of 85.2%, with a substantial agreement with computed tomography, indicating its accuracy in diagnosing and identifying the type and depth of fractures in infants with head trauma [47].

In summary, the comparability or superiority of POCUS to traditional imaging modalities across various applications underscores its significant value in neonatal care, offering rapid, accurate, and non-invasive diagnostic capabilities.

The burgeoning use of POCUS in neonatology presents an array of benefits, ranging from enhancing diagnostic precision to enabling rapid, bedside decision-making. However, this proliferation also brings to the forefront significant challenges in the education and training of physicians. The nuanced and delicate nature of neonatal care, coupled with the technical and interpretive skills required for proficient POCUS use, necessitates a tailored approach to training. The development of formal training requirements and evidence-based guidelines is essential for standardizing POCUS utilization across neonatal care centers. This review delves into these challenges, focusing on simulation-based training approaches, and outlines strategies for developing comprehensive training frameworks. Simulation-based training emerges as a pivotal strategy in overcoming educational barriers, offering a hands-on, risk-free environment where physicians can refine their ultrasound skills. The complexity of neonatal anatomy and the subtleties of pathological findings in this patient population demand a high level of skill and confidence from operators. Simulation allows for the replication of a wide range of neonatal conditions, from common pathologies to rare and critical scenarios, ensuring that physicians are well-prepared for real-world applications.

Key components of a simulation-based training approach include the following:

- High-Fidelity Simulators: Advanced simulators that mimic neonatal anatomy and pathology with high realism can significantly enhance learning outcomes. These simulators should offer a variety of clinical scenarios, allowing for comprehensive training in both common and complex POCUS applications in neonatology [55,86].
- Feedback and Assessment: Structured feedback mechanisms are vital for effective learning. Incorporating real-time feedback, guided by experienced instructors, helps in refining technique, improving diagnostic accuracy, and fostering clinical decision-making skills. Regular assessments, both formative and summative, are crucial for evaluating progress and competency [55,69,87,88,89].
- Integration of Clinical Reasoning: Simulation training should not only focus on technical skills but also integrate clinical reasoning and decision-making processes. This holistic approach ensures that physicians can effectively translate POCUS findings into actionable insights in clinical practice [12,90,91,92].

Alongside simulation-based training, other potential educational methods and course concepts, such as didactic sessions, lectures to introduce POCUS with its technical and physiological aspects, and bedside teaching during hands-on scanning are mentioned in the literature. During supervised hands-on training, instructors teach scanning skills and image interpretation competencies [71,93]. Theoretical knowledge can be assessed using pre- and post-tests. Practical skills can be assessed using objective structured clinical examinations (OSCEs) at course completion [94]. These strategies could complement simulation-based training and merit further investigation in future studies.

As the adoption of POCUS in neonatology accelerates, there is a pressing need to establish formal training requirements. These requirements should encompass both the technical aspects of ultrasound operation and the clinical interpretation of findings, tailored to the unique demands of neonatal care. Core competencies should include the following [95]:

- Understanding of neonatal anatomy and physiology as it pertains to ultrasound imaging.
- Mastery of ultrasound techniques, including probe handling, image acquisition, and optimization.
- Proficiency in the interpretation of ultrasound images, with the ability to distinguish normal from pathological findings.
- Integration of POCUS findings into clinical management plans.

Developing a standardized curriculum that encompasses these competencies is imperative. Such a curriculum should be adaptable, allowing for updates as new evidence emerges and technologies advance. Certification and credentialing processes, aligned with the curriculum, can help in maintaining high standards of practice and ensuring uniformity in POCUS proficiency across neonatal care providers. The establishment of evidence-based guidelines is critical for standardizing POCUS use across neonatal care centers. These guidelines should detail the indications for POCUS, outline standardized protocols for image acquisition and interpretation, and provide recommendations for the integration of POCUS findings into clinical care. Developing these guidelines requires a collaborative effort among neonatologists, radiologists, and other stakeholders, with a strong foundation in the latest research and best practices [8,24,36,96].

## 5. Limitations

The limitations of this systematic literature review on POCUS in neonatology include several key aspects. First, the review is limited by the scope of the literature it analyzes, which includes only published and accessible studies up to a specific cutoff date. This may exclude the most recent developments or ongoing research that could provide additional insights into the applications and effectiveness of POCUS. Additionally, the review restricts included studies to those published in English or German, potentially omitting valuable research conducted in other languages, which could lead to publication bias. Another significant limitation is the methodological variability among the included studies. These studies differ widely in terms of design, methodology, sample size, and quality. Such heterogeneity can complicate efforts to generalize findings across different settings and populations. The geographical variation in the implementation of POCUS training also poses a limitation. The review notes that POCUS training is suboptimally implemented in many countries, with notable differences between regions such as Europe and the USA. This variability can affect the generalizability of the results and the global applicability of the conclusions drawn from the review. Moreover, the steep learning curve associated with acquiring proficiency in POCUS is highlighted as a significant barrier. This complexity may limit the widespread adoption and effective use of POCUS, particularly in resource-limited settings where training opportunities and resources may be scarce. Finally, the inherent limitations of the included studies could affect the overall strength and reliability of the evidence. The review uses tools like the Medical Education Research Study Quality Instrument (MERSQI) to assess methodological quality, but the quality and strength of evidence from individual studies still influence the conclusions. Moreover, it is important to acknowledge that our review may contain some degree of redundancy due to the inclusion of both original studies and reviews. While we aimed to provide a broad overview of the applications of POCUS in neonatology, the inclusion of reviews may have led to overlap with the findings of original studies. No specific checks were conducted to ensure the exclusivity of information presented in reviews compared to original studies, which could have introduced bias. These limitations underscore the need for ongoing research, standardized training programs, and updates to clinical guidelines to ensure the effective and consistent use of POCUS in neonatal care. 

## 6. Future Research

Future research on POCUS in the neonatal intensive care unit is poised to explore various dimensions of its use, ranging from technology enhancements and educational strategies to protocol standardization and integration with other diagnostic modalities. Advancements in ultrasound technology might include tailoring developments to enhance resolution and diagnostic accuracy specifically for neonatal patients. The potential integration of artificial intelligence with POCUS could lead to automated interpretations and recommendations, streamlining diagnostics and therapeutic approaches in the NICU. Exploring the optimal ultrasound settings and probe types for different neonatal applications, such as the heart, lungs, and brain, is also vital. The clinical applications of POCUS could significantly impact the management of neonatal conditions, offering comparative benefits over traditional imaging methods like X-rays. Its efficacy in emergency scenarios, routine hemodynamic assessments, and the improvement of workflow and accuracy in procedures such as neonatal cardiopulmonary resuscitation are crucial research areas.

Educationally, identifying effective training frameworks and simulation models for neonatologists and NICU staff is essential, as is developing methods to assess POCUS competency across varying experience levels. On the procedural front, creating standardized protocols for global NICU implementation and adapting these protocols for low-resource settings are significant challenges.

Patient safety and quality improvement are also central themes, with research needed to assess the impact of POCUS on patient safety, the reduction of neonate transport risks, and the overall quality of care in the NICU. Furthermore, integrating POCUS with other diagnostic technologies like MRI or X-ray could enhance NICU diagnostic capabilities, necessitating investigations into the clinical benefits and limitations of such integrations.

Longitudinal and outcome-based research could examine the long-term impacts of POCUS on neurocognitive development, survival rates, NICU stay lengths, and hospital costs. These studies could help determine how POCUS effectiveness varies among different neonatal populations, including preterm and full-term neonates.

Ethical and regulatory considerations, including the potential biases linked to operator dependency and the certification of POCUS devices for neonatal use, warrant thorough investigation. Lastly, assessing the health economic aspects, such as the cost-effectiveness of POCUS compared to traditional imaging methods, is critical.

This comprehensive research agenda aims to harness the full potential of POCUS to not only enhance diagnostic precision but also improve patient outcomes and optimize technology use within the NICU setting. Each outlined question could lead to targeted studies that refine POCUS applications, ensuring they meet the unique needs of neonates in NICU care.

## 7. Conclusions

In conclusion, the integration of point-of-care ultrasound (POCUS) into neonatal care represents a significant advancement, offering the potential to revolutionize diagnostics and treatment strategies within this specialized patient population. The advantages of POCUS, including its non-invasiveness, immediacy, and versatility, align well with the critical and sensitive nature of neonatal care. However, the successful incorporation of this technology into clinical practice hinges on addressing several key challenges, notably in the realms of education and standardization. The development and implementation of robust, simulation-based training programs tailored specifically to neonatology are paramount [2,93,97,98]. Such programs, by simulating a wide array of clinical scenarios, can significantly enhance the proficiency of clinicians in using POCUS, ensuring that they are well-equipped to leverage this technology to its fullest potential. Additionally, while our review included five guidelines primarily focusing on shock protocols for crashing neonates, it is important to emphasize the need for comprehensive evidence-based guidelines that cover various aspects of POCUS application in neonatology [18,28,37,68]. These guidelines should encompass indications, protocols for image acquisition and interpretation, and the integration of ultrasound findings into clinical decision-making processes. The development of these guidelines should be a collaborative endeavor, engaging experts from various disciplines to ensure comprehensive and practical recommendations. As we move forward, it is crucial for the further POCUS implementation into neonatal care to recognize the potential benefits of POCUS while actively addressing the challenges associated with its integration. By investing in education, standardization, and research, we can harness the full potential of POCUS to enhance patient care, improve outcomes, and foster innovations in neonatal medicine. The future of neonatal care is promising, with POCUS poised to play a central role in its evolution. Ongoing technical developments and improvements in advanced imaging techniques, such as point-of-care magnetic resonance imaging, will shape the future of bedside imaging algorithms, including POCUS, in the NICU [99].

## Figures and Tables

**Table 1:** Study characteristics of included studies according to SPIDER.

Sample	Newborns, patients of neonatology
Phenomenon of Interest	Clinical presentations and indications requiring point-of-care ultrasound; applications of point-of-care ultrasound
Design	Review, prospective study, retrospective study, cross-sectional study
Evaluation	Point-of-care ultrasound
Research type	Qualtitative method

**Table 2:** Diagnostic and procedural applications of point-of-care ultrasound (POCUS) in neonatology.

**Diagnostic Applications**		
**Cardiac POCUS**	Global cardiac function	[2,8,12,13,14,15,16,17,18,19,20,21,22,23,24,25]
	Volume status	[2,12,14,19,20,23,24,26]
	Systemic blood flow	[19]
	Effect of inotropes on cardiac function	[16,26]
	Fluid responsiveness	[13,16,21,22,24]
	Persistent pulmonary hypertension	[2,14,17,18,19,22,23,24,26]
	Pericardial effusion	[2,8,12,13,14,16,19,20,21,22,23,24,27]
	Cardiac tamponade	[2,16,23,25,27]
	Atrial thrombus	[28]
	Assessment of inferior vena cava	[8,15,20,21]
	Unveiling pseudo-pulseless electrical activity in resuscitation	[29,30]
	Shock and hemodynamic instability	[15,22,24,30,31]
	Heart rate assessment	[32]
	Tetralogy of fallot	[33]
	Intracardiac shunts (patent ductus arteriosus, foramen ovale)	[17,18,19,21,22,24,26]
	Valvular dysfunction	[21,23]
	Transition of fetal to neonatal circulation	[34]
	Endocarditis	[24]
**Lung POCUS**	Pleural effusion	[2,8,14,15,17,18,20,21,22,24,25,26,31,35,36]
	Pneumothorax	[8,14,15,17,18,20,21,22,24,25,26,31,35,36,37,38,39]
	Pneumomediastinum	[35]
	Consolidation	[2,14,15,20,35,37,39]
	Further lung pathology (e.g., hyperinflation, atelectasis, air leak syndromes, oedema)	[12,15,18,20,22,24,31,36,38,39]
	Transient tachypnoea of the newborn	[2,21,22,24,35,36,40]
	Monitoring of surfactant therapy	[2,22,35,37]
	Neonatal acute respiratory distress syndrome	[2,8,18,21,22,24,35,37,38,39,41]
	Transient respiratory distress syndrome	[8,18,35]
	Meconium aspiration syndrome	[18,21,24,35,38]
	Congenital pneumonia	[14,15,18,21,22,24,35,36,38]
	Severity of bronchiolitis	[14,24,37,42]
	SARS-CoV-2 virus infection	[43]
	Prediction of bronchopulmonary dysplasia	[35]
	Congenital lung malformations, congenital diaphragmatic hernia, congenital chylothorax	[2,17,18,35]
	Diaphragmatic motion assessment	[2,21]
	Lung assessment during resuscitation	[35]
	Quantification of lung aeration	[14,35]
	Monitoring respiratory support	[8,14,35]
**Cranial POCUS**	Hemorrhage	[2,8,12,15,17,18,22,24,25,26,31,36,44]
	Periventricular leukomalacia	[26]
	Hydrocephalus	[8,12,15,18,22,45,46]
	Skull fracture	[47]
	Cerebral midline shift	[22,24]
	Cranial shunt failure	[48]
	Cerebral sinovenous thrombosis	[49]
	Cerebral blood flow	[2,14,17,18,22,24]
	Hypoxic ischemic encephalopathy	[18,24]
	Ocular ultrasound	[14]
	Optic nerve sheath diameter indicative of raised ICP	[2,24,48]
**Airway POCUS**	Vocal cord mobility	[12,21]
**Abdominal POCUS**	Major organ abnormalities	[15,24]
	Intraabdominal and pelvic free fluid	[2,15,22,24,25,31,36,50]
	Bowel/gut perforation	[24,31]
	Ascites	[2,8,24,36]
	Mesenteric blood flow/ischemia	[2,17,22,31]
	Neonatal ileus	[18,51]
	Necrotizing enterocolitis	[2,18,21,22,24,36,50,51]
	Meconium peritonitis	[51]
	Incarcerated inguinal hernia	[51]
	Intussusception	[52]
	Volvulus	[17,51]
	Renal blood flow	[17]
	Gastric emptying, content, and volume; risk assessment of pulmonary aspiration during sedation	[14,21,53,54]
	Guiding of enteral nutrition	[53]
	Ultrasound meal accommodation test	[53]
	Evaluation of gastrointestinal integrity	[24,53]
	Gastric foreign body investigation	[54]
	Hypertrophic pyloric stenosis diagnosis	[54]
	Gastric insufflation during mechanical ventilatory support	[54]
**Urogenital POCUS**	Bladder volume measurement	[8,14,22,36]
	Anuria	[18,22,36]
	Obstructive uropathy	[24]
**Vascular POCUS**	Deep vein thrombosis	[24]
	Acute critical aortic occlusion	[25]
	Vena cava thrombosis	[17]
**Emergency POCUS**	FOCUS: Focused cardiac ultrasound	[14]
	FAST: Focused assessment with sonography in trauma	[14,21]
	RANS: Rapid assessment of the neonate with sonography examination	[21]
**Procedural applications**		
	Neonatal endotracheal tube placement	[2,14,18,21,22,26,35,36,55,56,57,58,59,60]
	Prediction of difficult laryngoscopy and cricothyrotomy guidance	[21,57]
	Peripherally inserted central catheter line tip location	[2,12,14,18,19,22,23,24,26,27,31,36,38,61,62,63,64,65,66,67]
	Umbilical arterial and venous central catheter line tip location	[2,8,12,14,18,22,23,24,26,27,31,36,38,62,66,67,68,69,70,71,72]
	Peripheral arterial and venous line placement	[2,8,18,73]
	Peripheral intravenous extravasation injuries	[74]
	Lumbar puncture	[2,8,18,22,26,36,59]
	Regional and neuraxial blocks	[14]
	Placement of nasogastric and orogastric tube	[12,21,54,59]
	Pericardiocentesis, thoracocentesis, paracentesis	[2,12,18,22,23,24,35,59]
	Monitoring of extracorporeal membrane oxygenation	[14,75,76]
	Suprapubic bladder aspiration	[2,18,22,36,59,77]
	Urinary catheter visualization	[8]
	Abscess drainage	[24]

## Data Availability

Data available on request from the authors.

## Supplementary Materials

The following supporting information can be downloaded at: https://www.mdpi.com/article/10.3390/life14060658/s1, Table S1: Original studies considering POCUS in neonatology—systematic research by SPIDER scheme, Table S2: Reviews considering Point-of-care ultrasound (POCUS) in neonatology, Table S3: Guidelines considering Point-of-care ultrasound (POCUS) in neonatology.
