# Supplementary material for: Applications of Point-of-Care-Ultrasound in Neonatology: A Systematic Review of the Literature
Source: Life (Basel). 2024 May 22;14(6):658. doi: 10.3390/life14060658 (PMC11204601; doi:10.3390/life14060658)
Supplement: Supplementary file 1 [file life-14-00658-s001.zip › Supplementary Table S1.pdf]

Table S1. Original studies considering POCUS in neonatology – systematic research by SPIDER scheme

Abbreviations: POCUS: Point-of-care ultrasound, PIS: prospective interventional study, POS: prospective observational study, CSS: cross-sectional study, RS: retrospective study, CXR: chest x-ray

**Author/Year/Title**		**Sample**	**Phenomenon of Interest** (Indications and applications of POCUS)	**Design** (Study design)	**Evaluation** (Type of applicated POCUS)	**POCUS findings**	**Further Information**
Addepalli et al. [33], 2022	Point-of-Care Ultrasound Diagnosis of Tetralogy of Fallot Causing Cyanosis: A Case Report	5 months, male	Tetralogy of fallot causing dyspnea and cyanosis	Case report	Cardiac POCUS	A ventricular septal deficit with an overriding aorta and right ventricular hypertrophy	POCUS is an invaluable tool for evaluating the undifferentiated patient presenting with dyspnea, particularly when working in a limited-resource setting with limited prenatal cardiac screening, comprehensive echocardiography, and pediatric cardiology consultation services.
Ali et al. [55], 2020	Simulator-based ultrasound training for identification of endotracheal tube placement in a neonatal intensive care unit using point of care ultrasound	-- (intubation ultrasound simulator)	Neonatal endotracheal tube placement	PIS	POCUS n.sp.	Sonographic appearances of tracheal and esophageal intubation	A 3-h simulator-based training session in intubation POCUS appears to improve accuracy and time-to-interpretation with repeated simulator use.

Amer et al. [61], 2023	The utility of point-of-care ultrasound protocol to confirm central venous catheter placement in the preterm infant	Premature babies ≤ 30 weeks gestation	Central catheter tip location	CSS	POCUS n.sp.	Visualization of the line within the tight atrium or at the junction between the right atrium and the superior/inferior vena cava	POCUS demonstrates sensitivity of 83% and specificity of 96% in evaluation of central line tip position in relation to chest radiographs as a reference standard.
Ariff et al. [56], 2022	Diagnostic accuracy of point-of-care ultrasound compared to standard-of-care methods for endotracheal tube placement in neonates	348 neonates, mean 32.6 weeks gestation	Neonatal endotracheal tube placement	CSS	POCUS n.sp.	Sonographic appearances of tracheal and esophageal intubation	Diagnostic accuracy of POCUS compared with at least two standard-of-care methods demonstrated 99.7% sensitivity, 91% specificity, and 98.9% agreement. The median time required for POCUS interpretation was 3.0 seconds for tracheal intubation. POCUS is a rapid and reliable method of identifying ETT placement in neonates.
Ben Fadel et al. [26], 2019	Point of care ultrasound (POCUS) in Canadian neonatal intensive care units (NICUs): where are we?	-	Assessment of patent ductus arteriosus, persistent pulmonary hypertension, and volume status; monitoring of inotropes on cardiac function; cranial	CSS	POCUS n.sp.	n.sp.	Most directors and trainees agreed that incorporating POCUS into neonatal care offers great benefits to this patient population. A national neonatal POCUS curriculum

			ultrasound for intraventricular hemorrhage and periventricular leukomalacia; pleural effusion; pneumothorax; peripherally inserted central catheter line tip position; umbilical arterial and venous lines position; endotracheal tube position; lumber puncture				should be designed with clear goals, well-defined learning outcomes, effective teaching methods and appropriate assessment tools.
Boyar et al. [74], 2018	Point-of-Care Ultrasound Use in Neonatal Peripheral Intravenous Extravasation Injuries: A Case Series	10 neonates, 24 to 39 weeks gestation	Examination of peripheral intravenous extravasation injuries	Case series	POCUS n.sp.	- Extravasations with parenteral nutrition: hypoechoic fluid pockets, uneven shapes, elevation of epidermis, dermis, and subcutaneous tissue, separation of the tissue planes → more extensive fluid collections: hyperechoic collections of fat lobules, surrounded by septae ("fat-clumping" phenomenon, "cobblestoning")<br>- Peripheral IV extravasation of clear fluid: small hyperechoic areas of extravasated coagulated blood at the site where the blood vessel was injured	POCUS generates real-time data, allowing clinicians to evaluate the skin morphology quickly and with better precision. Measuring the epidermal-dermal-subcutaneous layer thickness together with the knowledge of the type of infusate allows improved indexing or scoring of the injury. This, in turn, may help guide therapy and follow-up.

						→ Larger extravasations of blood during transfusion: extensive hyperechoic regions surrounded by hypoechoic areas (pressure-related edema)	
Cowan et al. [62], 2021	Implementing point of care ultrasound in the neonatal intensive care unit: a safety study	50 neonates, mean 33 weeks gestation	Central catheter tip location (umbilical venous catheter, umbilical arterial catheter, peripherally inserted central catheter)	POS	POCUS n.sp.	n.sp.	Identifying central catheter tip location using POCUS in neonates appears safe without affecting cardiorespiratory stability (equivalence was demonstrated in HR, RR, and SpO2 before and after POCUS)
Dasani et al. [73], 2023	POCUS increases successful placement of peripheral arterial lines in neonates by less experienced providers	29 neonates, mean 37 weeks gestation	Success of peripheral arterial line placement	RS	POCUS n.sp.	Once the artery was identified via 2D and Doppler, the angiocatheter was inserted transcutaneously and advanced under ultrasound guidance using the out-of-plane approach until the catheter was visualized in the vessel with blood return. Once the catheter was confirmed to be in the vessel, the stylet was removed.	Use of POCUS for peripheral arterial line placement was associated with fewer attempts for successful placement in infants ≥ 2.5 kg compared to the traditional landmark-based approach
Davenport et al. [44], 2023	Brain Point of Care Ultrasound in Young Children Receiving Computed Tomography in the	21 newborns, mean age 3 weeks	Identify clinically significant brain injuries as defined by the Pediatric Emergency Care Applied Research	POS	Brain POCUS	- anterior fontanelle: serves as the necessary acoustic window → patent in more than 75% of infants at 12 months, only 10% of infants having a patent fontanel by the age of 2	The sensitivity of brain POCUS was 60% with a specificity of 94%. The diagnostic accuracy of brain POCUS was 86%.

	Emergency Department: A Proof of Concept Study		Network (PECARN) as traumatic brain injuries that require neurosurgical intervention, intubation, hospital admission, or result in death (e. g. intracranial bleeds)			→ limited views of the infratentorial structures including the cerebellum<br>- mastoid fossa: second acoustic window to examine fully the brain of the neonate	
Elsayed et al. [41], 2020	Lung ultrasound predicts histological lung injury in a neonatal model of acute respiratory distress syndrome	15 neonatal piglets, mean age 3 days	Acute respiratory distress syndrome	Case control study	Lung POCUS	n.sp.	POCUS may be valuable in neonates for early quantification of acute lung injury or ARDS
Grasso et al. [63], 2022	Ultrasound Guided Catheter Tip Location in Neonates: A Prospective Cohort Study	102 neonates, mean 31 weeks gestation	Central catheter tip location (peripherally inserted central catheter)	POS	POCUS n.sp.	B-mode views: apical 4-chamber view, left parasternal short axis view, bicaval subcostal view, and high right parasternal longitudinal view. tips positioned in the lower third of the superior vena cava, in the cavoatrial junction or in the right atrium.	Feasibility of POCUS guided tip location was 92.3% in our population. Failures were significantly associated with mechanical ventilation (OR 5.33)
Halm et al. [45], 2021	Evaluation of Ventricle Size Measurements in Infants by Pediatric Emergency Medicine Physicians	15 neonates, mean 28.9 weeks gestation	Identification of hydrocephalus	Pilot study	Cranial POCUS	Cranial POCUS through the open anterior fontanel to determine the size of the left and right ventricles by measuring the anterior horn width at the foramen of Monroe in coronal view.	The sensitivity and specificity of the pediatric emergency medicine (PEM) physicians performed cranial POCUS was 66.7% and 94.4%. The positive and negative

							predictive values were 88.9% and 81.0%. The interrater reliability between the PEM physician's and radiologist’s measurements was r = 0.91. The entire POCUS examinations performed by the PEM physicians took an average of 1.5 minutes.<br>While the PEM physicians in our study were able to determine the absence of hydrocephalus in infants with high specificity using cranial POCUS, there was insufficient evidence to support the use of this modality for identifying hydrocephalus.
Ibarra-Rios et al. [43], 2022	Lung Ultrasound Characteristics in Neonates with Positive Real Time Polymerase Chain Reaction for SARS-CoV-2 on a Tertiary Level	32 neonates, median 36 weeks gestation	Acute respiratory syndrome secondary to SARS-CoV-2 virus infection	CSS	Lung POCUS	Abnormalities found were B-lines, interstitial pattern, irregular/interrupted/thick pleural line, small (≤5 mm) and extensive consolidations (≥5 mm). Consolidations showed posterior predominance.	POCUS is useful in assessing lung involvement that correlated with the degree of disease severity and respiratory support (FiO2 needed, increase in positive end expiratory pressure)

	Referral Hospital in Mexico City						
Ismail et al. [38], 2023	Lung Ultrasound Role in Diagnosis of Neonatal Respiratory Disorders: A Prospective Cross-Sectional Study	100 neonates, gestational age ≥28 weeks	Neonatal respiratory disorders (respiratory distress syndrome, pneumonia, meconium aspiration syndrome, pneumothorax, and pulmonary atelectasis)	CSS	Lung POCUS	Lung POCUS score:<br>0: Denotes A-pattern (A-lines only, which emerges from the pleural line reverberation artifact);<br>1: B-pattern (≥3 well-spaced B-lines; B-lines are lines reaching the screen edge in the absence of fading);<br>2: Severe B-pattern (coalescent and crowded B-lines with or without consolidations restricted to the subpleural space);<br>3: Extended consolidations.	The total agreement between lung POCUS and CXR was 98.5%, leading to considerable agreement in the diagnosis of neonatal respiratory disorders. Lung POCUS is a reliable bedside modality of diagnosis and is safer than CXR and should be considered an alternative method in diagnosis.
Klimek et al. [28], 2023	Spontaneously disappearing right atrial mass in a preterm infant: a case report	Neonate, 25 weeks gestation	Atrial thrombus	Case report	Cardiac POCUS	Large pedunculated echogenic mass in the right atrium	POCUS may help to diagnose cardiac conditions that may otherwise have been missed or delayed where echocardiography may not be available or not clinically indicated.
Kochan et al. [69], 2021	Point-of-Care Ultrasound to Confirm Umbilical Line Placement	-	Umbilical central catheter tip location	POS	POCUS n.sp.	-	To increase the use of POCUS in neonatal practice, neonatal training programs should consider incorporating phantom models in their POCUS curriculum.

Lin et al. [64], 2023	Point-of-care ultrasound (POCUS) for tip localization of neonatal peripherally inserted central catheter (PICC): A prospective study	63 neonates, median 31 weeks gestation	Peripherally inserted central catheter (PICC) line tip location	POS	POCUS n.sp.	Subclavicular and subxiphoid sagittal view radiography:<br>- optimal catheter tip position: in the superior or inferior vena cava proximal to the cavoatrial junction but not entering the right atrium<br>-too deep insertion of the tip: cardiac arrhythmia, perforation, pericardial effusion, and cardiac tamponade<br>- too shallowly insertion in noncentral veins (internal jugular, subclavian, ascending lumbar, superficial abdominal, and renal vein): decreased hemodilution, extravasations, seizures or paraplegia, thrombosis, and death	POCUS during neonatal PICC insertion can significantly improve the likelihood of optimal tip position. By enabling real-time adjustments, POCUS helps to minimize the need for repetitive repositioning, thereby reducing radiation exposure
Mahdipour et al. [77], 2021	Strengthening the success rate of suprapubic aspiration in infants by integrating point-of care ultrasonography guidance: A parallel-randomized clinical trial	114 neonates and infants with suspected urinary tract infection	Sterile collection of urine samples using suprapubic aspiration	POS	POCUS n.sp.	A linear US probe was used to assess the presence of adequate urine and visualize the needle's tip in the path of real-time aspiration.	Results showed that the POCUS-SPA had the highest success rate (97.4 %) in urine sampling, compared with Non-POCUS-SPA and bladder catheterization.

Manzar and Bhat [32], 2022	Feasibility of handheld ultrasound to assess heart rate in newborn nursery	30 neonates, median 39.1 weeks gestation	Heart rate (HR) assessment during neonatal resuscitation	POS	Cardial POCUS	Counting of the heart beats for six seconds, multiplied by ten	The most reliable ways of assessing HR at birth are pulse oximetry and electrocardiograph, but delayed recording time due to poor perfusion or probe and electrode placement are concerns. POCUS provides a feasible real-time instant view of the heart to assess the HR of stable newborn infants.
Motz et al. [65], 2019	Point-of-care ultrasound for peripherally inserted central catheter monitoring: a pilot study	30 neonates, mean 29.4 weeks gestation	Peripherally inserted central catheter line tip location	POS	POCUS n.sp.	Upper extremity: three B-mode ultrasound views:<br>- subclavicular view of the anterior chest: PICCs in the subclavian or brachiocephalic veins<br>- right parasternal sagittal view: superior vena cava (SVC)<br>- subcostal long view: rule out pericardial effusion and evaluation for PICCs in the lower SVC/right atrium and right ventricle<br><br>lower extremity: two B-mode ultrasound views:<br>-right-sided flank view: inferior vena cava	This study demonstrates the feasibility and accuracy of using POCUS for PICC monitoring in the NICU. (sensitivity of 0.97, specificity of 0.66 and positive predictive value of 0.98)

						- subcostal long view: assess for PICCs in the right atrium/ventricle	
Oulego-Erroz et al. [49], 2020	Point-of-care ultrasound in the diagnosis of neonatal cerebral sinovenous thrombosis	27 days old neonate	Neonatal cerebral sinovenous thrombosis (CSVT)	Case report	Cranial POCUS	-Thrombus within the transverse sinuses appearing as an echogenic material filling the sinus lumen<br>-Absence or reduction of flow within the thrombosed sinuses on color Doppler imaging<br>-Potential identification of thrombus in other dural venous sinuses such as the sigmoid sinus	This case report highlights how cranial POCUS allowed neonatologists to promptly diagnose CSVT in a newborn with complex clinical presentation, by directly visualizing thrombus within the transverse sinuses on ultrasound images obtained through the mastoid fontanelle. Timely diagnosis facilitated urgent treatment of this severe form of neonatal stroke.
Pai et al. [8], 2022	Implementation of a Bedside Point-of-Care Ultrasound Program in a Large Academic Neonatal Intensive Care Unit	-	Umbilical line visualization, cardiac, lung, abdomen (including bladder), and cranial as well as procedural applications	POS	POCUS n.sp.	-Umbilical line assessment (Visualization, Placement)<br>-Cardiac (Function, Inferior vena cava filling, Pericardial effusion)<br>-Lung (Effusion, Pneumothorax, Evaluation of lung pathology while on respiratory support (respiratory distress syndrome, transient tachypnea of the newborn))	POCUS guided therapy and influenced clinical management in all applications. Implementation of a POCUS program is feasible in a large academic NICU and can benefit from a team approach.

						- Cranial (intraventricular hemorrhage, Ventricular size assessment)<br>-Abdomen/Bladder (Bladder volume assessment, Urinary catheter visualization, Ascites)<br>-Lumbar puncture<br>-Peripheral vascular access	
Parri et al. [47], 2018	Point-of-Care Ultrasound for the Diagnosis of Skull Fractures in Children Younger Than Two Years of Age	115 children, mean age 7.6 months	Diagnosis of skull fractures	POS	Skull POCUS	-true positive fracture: cortical skull irregularities visible in multiple orientations (contralateral skull area was used for comparison to differentiate these from suture lines)<br>-linear, depressed, or complex<br>-Fracture size: measuring the outer cortices of the 2 fracture fragments.<br>-Fracture depth: ≤3 mm, 4-6 mm, 7-10 mm, or ≥11 mm	POCUS had a sensitivity of 90.9 % and a specifity of 85.2 % for identifying skull fractures.<br>Agreement between POCUS and CT to identify the type of fracture was 84.4%.<br>POCUS may identify the type and depth of fractures in infants with local physical signs of head trauma with substantial accuracy.
Pawlowski et al. [76], 2021	Point-of-care ultrasound for the evaluation of venous cannula position in neonatal extracorporeal membrane oxygenation	37 neonates, mean 38.6 weeks gestational age	Identify venous cannula position in neonates on extracorporeal membrane oxygenation	RS	POCUS n.sp.	n.sp.	This study provides preliminary evidence that POCUS is more accurate than plain radiography for the evaluation of ECMO venous cannula position. Adoption of this practice may prevent potentially catastrophic ECMO complications.

Pershad et al. [48], 2017	Imaging Strategies for Suspected Acute Cranial Shunt Failure: A Cost-Effectiveness Analysis	n.sp.	Evaluate suspected acute cranial shunt failure/raised ICP	n.sp.	optic nerve POCUS	Optic nerve sheath diameter (ONSD) measured POCUS may have utility as a rapid bedside screening test for elevated intracranial pressure (ICP)	For patients with low pretest probability of shunt failure and a normal shunt series (plain radiograph), a screening measurement of optic nerve sheath diameter is the most cost-effective approach.
Polonio et al. [71], 2022	Implementation of point of care ultrasound to assess umbilical venous catheter position in the neonatal intensive care unit	185 neonates	Umbilical central catheter tip location	POS	POCUS n.sp.	The umbilical venous catheter (UVC) tips are visualized in the inferior vena cava.	A training module for POCUS proficiency in assessing UVC position in neonatal intensive care unit was implemented. The median time to achieve proficiency was 5 months. A minimum of two views (subcostal and parasternal short view) were required to correctly assess catheter tip location.
Rossi et al. [66], 2022	Point-of-care ultrasound for neonatal central catheter positioning: impact on X-rays and line tip position accuracy	144 neonates, mean 32 weeks gestation	Central catheter tip location (umbilical and peripherally inserted)	RS	POCUS n.sp.	Tip seen in cavoatrial junction	POCUS used for line tip positioning in the NICU significantly decreased the number of X-rays performed and was associated with better-positioned lines, decreased irradiation and cost

San Sebastian Ruiz et al. [42], 2021	Point-of-care lung ultrasound in children with bronchiolitis in a pediatric emergency department	200 children, mean age 5.7 months	Assessment of bronchiolitis and need for respiratory support	POS	Lung POCUS	Bronchiolitis: not specific, patchy interstitial pattern with subpleural consolidations<br>Classification of lung findings:<br>-normal pattern (A): hyperechoic pleural line sliding with breathing (well defined, regular, and dynamic), horizontal artifacts under the pleural line (A-lines: hyperechoic, static, and equidistant from each other, indicating the normal presence of air)<br>-moderate interstitial pattern (B1), severe interstitial pattern (B2): vertical lines (B-lines: hyperechoic, well defined, erasing A-lines, moving with the pleural line),<br>white lung pattern<br>-isolated consolidation (C): Areas of pulmonary hypoventilation (absence of alveolar air, adjacent to the pleural line, poorly defined contours with hyperechoic images inside the consolidation (air bronchogram))	Moderate bronchiolitis is correlated with abnormal lung POCUS. The severity of interstitial ultrasound pattern has some correlation with an increased need for respiratory support.
Sosa et al. [50], 2024	Point-of-care ultrasound evaluation of suspected	Male newborn, 6 weeks old	Evaluation of suspected necrotizing enterocolitis	Case report	Abdominal POCUS	Pneumatosis intestinalis (echogenic foci within the bowel wall), pneumoperitoneum, free	In our case, the use of POCUS expedited and increased our clinical suspicion for NEC in a

	necrotizing enterocolitis in the ED					peritoneal fluid, portal venous gas, ileus	seemingly non-toxic infant. This enabled escalation of care prior to the development of more severe symptoms, and a potentially poor outcome was avoided.
Xie et al. [72], 2023	Point-of-care ultrasound for monitoring catheter tip location during umbilical vein catheterization in neonates: a prospective study	68 neonates, mean 33.4 weeks gestation	Umbilical central catheter tip location	POS	POCUS n.sp.	The umbilical venous catheter (UVC) tips are visualized in the cavoatrial junction	The catheter tip is prone to displacement and misalignment after umbilical vein catheterization, which most commonly occurs on days. POCUS is recommended for daily monitoring of the tip location during umbilical vein catheterization until catheter removal.
Zaghloul et al. [67], 2019	The superiority of point of care ultrasound in localizing central venous line tip position over time	56 neonates	Central catheter tip location (umbilical and peripherally inserted)	POS	POCUS n.sp.	Tip seen in cavoatrial junction	Agreement between POCUS and X-rays for central venous catheter tip position was high, with the highest in birth weight < 1000 g. The data suggest that POCUS can be used for initial confirmation and follow up of tip position.
Zaytseva et al. [60], 2020	Determination of optimal endotracheal tube tip (ETT) depth	75 neonates, median 3 days of age	Optimal endotracheal tube tip depth	POS	POCUS n.sp.	Using ultrasound to determine optimal ETT tip depth:<br>A 10 MHz cardiac probe was used in the high parasternal	Ultrasound measurements of ETT tip location showed substantial agreement

	from the gum in neonates by X-ray and ultrasound					view to visualize the trachea and mainstem bronchi and to measure the distance from the ETT tip to carina level. A regression equation was derived based on CXR measurements of optimal ETT tip location relative to weight: Optimal depth from gum (cm) = 5.21 + 1.03 x weight (kg)	with CXR (intraclass correlation 0.95). Ultrasound provides a rapid, non-invasive method to confirm optimal ETT placement in the mid-trachea at the carina level in neonates.
Zhang et al. [27], 2023	The advantage of point-of-care ultrasound in central venous catheterization and related pericardial effusion in infants in the NICU	25 neonates, mean 29.3 weeks gestation	Detection of central venous catheterization (CVC) related pericardial effusion (PCE)	RS	Cardiac POCUS	Cardiac POCUS showed that the catheter tip extended deep into the heart in 72% of infants with PCE. The advocate placement of the peripherally inserted catheter tip is the intersection of the superior vena cava and right atrium. The advocate placement of the umbilical inserted catheter tip is the intersection of the inferior vena cava and right atrium.	Compared with chest radiography, POCUS is more accurate for central catheter tip positioning and can detect PCE more quickly. Early identification and diagnosis can effectively reduce fatality rates and improve the prognosis of infants with CVC-associated PCE.
