# Supplementary material for: Applications of Point-of-Care-Ultrasound in Neonatology: A Systematic Review of the Literature
Source: Life (Basel). 2024 May 22;14(6):658. doi: 10.3390/life14060658 (PMC11204601; doi:10.3390/life14060658)
Supplement: Supplementary file 1 [file life-14-00658-s001.zip › Supplementary Table S2.pdf]

Table S2. Reviews considering Point-of-care ultrasound (POCUS) in neonatology

**Author/year/title**		**Applications of POCUS in neonatology**
Arnold et al. [12], 2023	Advanced imaging and modeling in neonatal simulation	- Lung: identify lung pathology (hyperinflation, atelectasis, fluid, and air leak syndromes) using beam reflection from the air-fluid interfaces in the lung interstitium and pleura<br>- Head: diagnose intracranial processes (intraventricular hemorrhage, parenchymal hemorrhage, and hydrocephalus)<br>- Larynx: vocal fold mobility issues and other subglottic airway issues<br>- Abdomen: free fluid, bowel integrity (e.g., perforation), necrotizing enterocolitis, guidance of paracentesis<br>- Cardiac: evaluation of global cardiac function and fluid status, central catheter tip location (e.g. umbilical venous catheter or percutaneous intravenous catheter), identification of pericardial effusion, and guidance for performing emergent pericardiocentesis<br>- Procedural: guided visualization during emergent invasive neonatal procedures (thoracentesis, pericardiocentesis, and paracentesis), guide vascular access, identify endotracheal tube location, lumbar puncture, and transpyloric feeding tube
Bhoil et al. [40], 2021	Signs and lines in lung ultrasound	- Transient tachypnoea of the newborn: sonographic difference in lung echogenicity between the upper and lower lung fields<br>➔ double lung point sign: refers to a sharp boundary found between relatively aerated (normal) superior lung fields and coalescent "B-lines" (representing interstitial edema) in the lower lung fields (this sign is very specific for TTN (with reported specificity of up to 100%) and is not seen in normal lungs, atelectasis, pneumothorax, pneumonia, or pulmonary hemorrhage)
Bianzina et al. [75], 2024	Use of point-of-care ultrasound (POCUS) to monitor neonatal and pediatric extracorporeal life support	- POCUS is an essential tool to monitor all phases of neonatal and pediatric ECMO: evaluation of ECMO candidacy, ultrasound-guided ECMO cannulation, daily evaluation of heart and lung function and brain perfusion, detection and management of major complications, and weaning from ECMO support<br>- Combined advances in ECMO technology and bedside medical management have improved general outcomes
Burton and Bhargava [57], 2023	A Scoping Review of Ultrasonographic Techniques in the Evaluation of the Pediatric Airway	- Endotracheal tube (ETT) sizing, ETT placement and depth confirmation, vocal fold assessment, prediction of post-extubation stridor, difficult laryngoscopy prediction, and cricothyrotomy guidance
Chan et al. [29], 2023	Unveiling pseudo-pulseless electrical activity (pseudo-PEA) in ultrasound-	- POCUS benefits cardiac arrest management, particularly in distinguishing true Pulseless Electrical Activity (PEA) from pseudo-PEA<br>- Pseudo-PEA is when myocardial motion can be seen on ultrasound but fails to generate palpable pulses or sustain circulation despite evident cardiac electrical activity

	integrated infant resuscitation	➔ Existing neonatal resuscitation protocols lack directives for identifying and effectively leveraging pseudo-PEA insights in infants, limiting their potential to enhance outcomes
Chidini and Raimondi [37], 2023	Lung ultrasound for the sick child: less harm and more information than a radiograph	- Lung Ultrasound (LUS) is useful in confirming diagnoses of pneumothorax, consolidation, and pleural effusion.<br>- LUS has demonstrated effectiveness in monitoring the response to surfactant therapy in neonates, in staging the severity of bronchiolitis, and in pediatric acute respiratory distress syndrome.
Congedi et al. [58], 2022	Sonographic Evaluation of the Endotracheal Tube Position in the Neonatal Population: A Comprehensive Review and Meta-Analysis	- Endotracheal intubation is a common procedure in both neonatal intensive care units patients facing respiratory failure, and in emergency settings like neonatal resuscitation<br>➔ Endotracheal intubation in neonates is challenging and requires a high level of precision, due to narrow and short airways, especially in preterm newborns. The current gold standard for endotracheal tube verification is chest X-ray. POCUS appears to be a fast and effective technique (sensitivity of 93.44%) to identify correct endotracheal intubation in newborns.
Conlon et al. [13], 2024	Cardiac point-of-care ultrasound: Practical integration in the pediatric and neonatal intensive care settings	- Cardiac POCUS to identify real-time hemodynamic pathophysiology<br>➔ Is my patient fluid-responsive?<br>➔ Is there a pericardial effusion and, within the clinical context, is there concern for hemodynamic significance?<br>➔ What is the right ventricular (RV) function?<br>➔ What is the left ventricular (LV) function?<br>➔ Is the RV dilated?<br>➔ Does the RV demonstrate evidence of systemic or suprasystemic pressure overload?
D'Andrea et al. [68], 2021	Umbilical Venous Catheter Update: A Narrative Review Including Ultrasound and Training	- The umbilical venous catheter (UVC) is one of the most commonly used central lines in neonates<br>➔ Providing stable intravenous access in infants requiring advanced resuscitation in the delivery room or needing medications, fluids, and parenteral nutrition during the 1st days of life<br>➔ UVCs are easy to insert, however when the procedure is performed without the use of ultrasound, there is a quite high risk, up to 40%, of non-central position<br>➔ The use of POCUS reduces catheter malpositioning and associated complications<br>➔ The subcostal longitudinal view is the most commonly used acoustic window, which allows staff to visualize the inferior vena cava and the right atrium, which are the targets. The tip is followed until it reaches the target zone.
Fernàndez et al. [35], 2022	Usefulness of lung ultrasound in the diagnosis and follow-up of respiratory diseases in neonates	Lung POCUS in neonatal care<br>- Pneumothorax: absence of lung sliding, absence of B lines, exclusive presence of A-lines behind sternum, lung point, stratosphere sign (M-mode)

		- Pneumomediastinum: depending on the size large A-line artefacts or small hyperechoic lines in the lateral margins of the thymus or in the thymic parenchyma (suprasternal plane)<br>- Lung consolidation: absence of pleural line, alveolar collapse with bronchogram, hepatization, shred sign<br>- Pleural effusion: anechoic area between parietal and visceral pleura, jellyfish sign (2D-mode), sinusoid sign (M-mode), quad sign (2D-mode)<br>- Transient tachypnoea of the newborn: Heterogeneous pattern with variable involvement in different areas, with: normal lung pattern, interstitial pattern, alveolar-interstitial pattern, double lung point sign (50%); mild changes in pleural line; mild pleural effusion<br>- Neonatal respiratory distress syndrome: homogeneous alveolar-interstitial pattern, irregular or thickened pleural line, consolidations of variable size: small subpleural collapses or atelectasis, subclinical pleural effusion<br>- Transient respiratory distress: variable pattern with alveolar-interstitial (no consolidations), minimal air leaks, or normal pattern<br>- Meconium aspiration syndrome: patchy pattern with areas of consolidations with air bronchogram (bilateral, variable size), alveolar-interstitial pattern or normal pattern; pleural effusion; dynamic features<br>- Congenital pneumonia: nonspecific pattern, varies depending on severity: interstitial pattern and/or alveolar-interstitial with consolidations, irregular thickened pleural line, subclinical pleural effusion<br>- Neonatal resuscitation: assessment of lung aeration at birth, monitoring the presence of lung sliding, monitoring alveolar recruitment during ventilation maneuvers, and detect potential complications during resuscitation, monitoring heart rate during cardiopulmonary resuscitation<br>- Prediction of surfactant administration<br>- Monitoring and detection of complications in patients receiving respiratory support: consolidation indicative of endotracheal tube malposition, ventilator-associated pneumonia, edema/extravascular lung fluid in neonates with poor lung clearance or congenital heart disease<br>- Changes in respiratory support: failure of non-invasive ventilation and weaning of invasive mechanical ventilation<br>- Ultrasound-guided procedures: thoracocentesis, verification endotracheal tube placement<br>- Congenital lung malformations<br>- Congenital diaphragmatic hernia<br>- Congenital chylothorax<br>- Monitoring of lung development in premature infants: prediction of bronchopulmonary dysplasia<br>- LUS performed between 60 and 120 mins post birth in term and late preterm neonates is a highly accurate predictor of NICU admission

Flores et al. [14], 2023	Point-of-Care-Ultrasound in Pediatrics: A Review and Update	- POCUS by pediatric intensivists<br>➔ Respiratory critical care: quick evaluation of pneumonia, atelectasis, pneumothorax, and bronchiolitis; quantifying lung aeration, identifying consolidations, and detecting pleural effusions; monitoring changes in lung pathology over time and assessing the response to treatment<br>➔ Cardiac critical care: cardiac arrest (identification of reversible causes of cardiac arrest including pericardial effusion, pneumothorax, intravascular volume depletion, right or left ventricular systolic dysfunction, etc.), extracorporeal membrane oxygenation (ECMO) assessment (identifying relevant anatomy and the potential for cannulation failure; guide peripheral, cervical (jugular), and femoral cannulation; assure correct tip position in e.g. in the right atrium/proximal inferior vena cava; identifying the return jet towards the tricuspid valve in case of venovenous ECMO cannulation with dual-lumen cannula; Functional examination of the failing heart in ECMO: progressive left heart failure in the absence of preload venting, examination of complications such as pneumothorax and effusions, insufficient and excessive loading conditions, cannula displacement)<br>➔ Neurologic care: visualization of the brain and ventricular spaces from the anterior fontanelle and mastoid windows; transcranial doppler (visualization of the anterior cerebral artery, middle cerebral arteries, and posterior circulation (flow velocity, resistance)), ocular ultrasound (identifying ocular movement after trauma to exclude entrapment, retinal integrity, and identification of the optic nerve)<br>- POCUS by pediatric cardiologists<br>➔ Pediatric cardiac catheterization: guidance for central venous and arterial access, avoidance of bleeding complications (pericardial effusion and tamponade)<br>➔ Lung POCUS: pulmonary congestion or edema in patients with heart disease)<br>- POCUS by pediatric anesthesiologist<br>➔ Regional and neuraxial blocks; central line placement, investigation of intra-operative hemodynamic comprise; endotracheal tube placement; optimization of pulmonary mechanics (Reducing atelectasis, identifying pleural effusion); gastric POCUS to reduce pulmonary aspiration<br>- POCUS by pediatric emergency physicians<br>➔ Focused cardiac ultrasound (FOCUS) (interrogate hemodynamic function, presence or absence of pericardial effusion, qualitative chamber size and systolic pressure); focused assessment with sonography in trauma (FAST) (pediatric thoracoabdominal trauma with hemoperitoneum, hemopericardium, hemothorax, and pneumothorax); soft tissue evaluation (abscesses, cellulitis, foreign bodies); bladder volume measurement; procedural guidance for vascular access (direct visualization of needle insertion)

		- Advantages of POCUS: rapid and non-invasive insights into structure and function of the pediatric heart/lung, facilitating early diagnosis of congenital or acquired abnormalities → timely interventions, prevention of complications, and improving patient outcome
Inarejos Clemente et al. [51], 2023	US for Evaluation of Acute Abdominal Conditions in Neonates	Abdominal POCUS can help, complement, and sometimes replace radiographic or contrast-enhanced studies in critically ill and labile neonates who are difficult to transport to the fluoroscopy suite.<br>- Neonatal ileus resulting in bowl distension (thinning of bowel wall thickness less than 1 mm, loss of normal layering of the bowel (hyperechogenicity of mucosa and muscularis propria, thickened parallel valvulae conniventes resulting in a "herringbone" or "zebra" pattern), decreased peristalsis)<br>- Necrotizing enterocolitis and its complications (intramural gas or pneumatosis (hyperechoic granular pattern of the bowel wall, with posterior reverberation artifacts, which does not change with peristalsis), portal venous gas (intravenous echogenic foci moving with the blood flow), pneumoperitoneum (peritoneal stripe sign), loss of normal layering of the bowel (hyperechogenicity of mucosa and muscularis propria, thickened parallel valvulae conniventes resulting in a "herringbone" or "zebra" pattern), free peritoneal fluid, thickening and secondary thinning of bowel wall (due to inflammation, ischemia, and necrosis; CAVE perforation), hyperemia (y-pattern, ring-pattern) and secondary decrease of vascularization due to necrosis)<br>- Malrotation with a midgut volvulus (relationship between small mesenteric arteria and vein, distal duodenum obstruction, proximal duodenum dilatation, non-retroperitoneal position of the third portion of the duodenum (bird's beak morphology), whirlpool sign)<br>- Segmental intestinal volvulus unrelated to malrotation (normal orientation of small mesenteric arteria and vein, normal retroperitoneal course of the third portion of the duodenum, normal midgut rotation, whirlpool-sign, distal bowel obstruction, proximal dilated fluid-filled loops of bowel, duplication cysts and meconium pseudocysts as a cause of volvulus)<br>- Meconium peritonitis (resulting from utero bowel perforation; antenatal US: fetal ascites, dilatation of intestinal loops, intraabdominal calcifications, echogenic bowel, polyhydramnios, and pseudocysts; postnatal US: proximal to inspissated meconium dilated bowel, distal dilated bowel, homogenous and anechoic ascites with internal septa, snowstorm appearance in patients with perforation, meconium pseudocyst, pneumoperitoneum)<br>- Incarcerated inguinal hernia (segment of intestine with wall thickening and hyperechogenicity, Color Doppler US and microvascular US help in the differentiation of incarcerated and strangulated hernias (absence of internal vascularity))
Jain et al. [16], 2023	Clarification of boundaries and scope of cardiac POCUS	Cardiac POCUS:<br>- Basic, time-sensitive, and focused ultrasound assessment of the heart to assist in urgent or emergent clinical decision-making and guide resuscitative interventions

	vs. Targeted Neonatal Echocardiography	- Aimed to answer problem-oriented clinical questions using discrete outcome measures, that complement clinical data and facilitate management of infants with "normal" cardiac anatomy<br>➔ Cardiac tamponade and pericardial effusion (+ following pericardiocentesis)<br>➔ Guiding evaluation of global cardiac function vs. cardiac filling status in acute cardiovascular collapse (underfilled heart (decreased preload, fluid therapy) or volume overload (increased preload, inotrope therapy)<br>➔ Umbilical venous catheter and central lines position (confirming catheter tip position in great veins)<br>➔ !! "not" to appraise and target management of specific physiologic states (pulmonary hypertension, patent ductus arteriosus), and gradation of cardiac dysfunction ➔ targeted neonatal echocardiography
Maddaloni et al. [17], 2023	The role of point-of-care ultrasound in the management of neonates with congenital diaphragmatic hernia	Management of neonates with a congenital diaphragmatic hernia<br>- The use of POCUS should be encouraged to improve ventilation strategies, systemic perfusion, and enteral feeding, and to intercept any early signs related to future neurodevelopmental impairment.
Meinen et al. [70], 2020	Point-of-care ultrasound use in umbilical line placement: a review	POCUS was shown to be feasible and appears to be a superior imaging modality to x-rays in assessing umbilical line tip location. POCUS is more accurate in determining umbilical catheter positioning, allows for more rapid line umbilical catheter placement, and reduces the time to treatment as well as radiation exposure to the neonate when compared with x-ray. The available studies support further education in training neonatal providers to become proficient in POCUS for assessing umbilical lines.
Miller et al. [18], 2020	Point-of-care ultrasound in the neonatal ICU	Diagnostic applications for POCUS in the NICU:<br>- Pulmonary:<br>➔ Pneumothorax: common neonatal complication, particularly in the setting of positive pressure ventilation; absent or diminished lung sliding, "lung point sign", absent B-lines, and absent lung pulse in the area of pneumothorax<br>➔ Pleural effusion: anechoic hypoechoic collection between the parietal and visceral pleura. Ultrasound allows quantification and monitoring of effusion size<br>➔ Respiratory distress syndrome: consolidation, bilateral "white lung" appearance, air bronchograms, pleural line abnormalities, and absent A-lines<br>➔ Transient tachypnea of the newborn: "double lung point" sign, pulmonary edema seen as B-lines/alveolar-interstitial syndrome, compact B-lines, and a regular pleural line without subpleural consolidation<br>➔ Pneumonia: areas of consolidation with irregular margins, dynamic air bronchograms within consolidations, and pleural line abnormalities

		➔ Pulmonary hemorrhage: "shred sign" of irregular interface between aerated and consolidated lung, consolidations with air bronchograms, pleural line abnormalities, and disappearing A-lines<br>➔ Meconium aspiration syndrome<br>➔ Diaphragm dysfunction<br>➔ Atelectasis<br>- Cardiac:<br>➔ Hemodynamic assessment: contractility, cardiac filling, cardiac output, superior vena cava flow; these parameters help evaluate causes of hypotension in neonates<br>➔ Patent ductus arteriosus: using ductal diameter measurements, markers of increased pulmonary flow like tricuspid regurgitation, and signs of diastolic steal<br>➔ Persistent pulmonary hypertension of the newborn: estimating pulmonary artery systolic pressure, direction of intracardiac and extracardiac shunts, ventricular size, and function assessment<br>- Abdominal:<br>➔ Necrotizing enterocolitis: pneumatosis intestinalis (intramural air in the bowel wall), free air, portal venous gas<br>➔ Intestinal dysmotility<br>➔ Anuria<br>- Neurology: using cranial ultrasound through the fontanelles in neonates<br>➔ Intraventricular hemorrhage<br>➔ Hypoxic ischemic encephalopathy<br>➔ Cerebral perfusion (Doppler)<br>➔ Hydrocephalus<br><br>Procedural:<br>- Peripheral intravenous lines<br>➔ Peripherally inserted central catheters<br>➔ Umbilical venous and arterial catheters<br>➔ Central venous catheters (internal jugular, subclavian, brachiocephalic, or femoral)<br>➔ Peripheral arterial catheters<br>- Endotracheal tube localization<br>- Lumbar puncture: Ultrasound identifies spinal anatomy and guides needle placement, improving success rates and decreasing traumatic taps.<br>- Fluid drainage

		➔ Thoracentesis<br>➔ Paracentesis<br>➔ Pericardiocentesis<br>- Suprapubic bladder aspiration<br><br>As the use of this tool increases, there is an urgent need to develop formal training requirements specific to neonatology, as well as evidence-based guidelines to standardize use across centers.
Nestaas [19], 2022	Neonatologist Performed Echocardiography for Evaluating the Newborn Infant	- Assessment of intravascular fluid volume and systemic blood flow:<br>➔ Measurements of blood flow velocity and cross-sectional areas over the aortic and pulmonary valves can be used to calculate systemic blood flow<br>➔ Superior vena cava flow measurement provides an estimate of cerebral blood flow<br>- Evaluation of blood pressure and vascular resistance in the pulmonary circulation:<br>➔ Tricuspid regurgitation velocity can be used to estimate systolic pulmonary artery pressure<br>➔ Pulmonary arterial acceleration time (PAAT)/right ventricular ejection time (RVET) ratio decreases with increasing pulmonary vascular resistance<br>➔ Ventricular septal position and shape provide information on diastolic and systolic pressures<br>- Assessment of ventricular function:<br>➔ Shortening fraction, ejection fraction from cavity changes<br>➔ Tricuspid annular plane systolic excursion (TAPSE), mitral annular plane systolic excursion (MAPSE) from tissue Doppler<br>➔ Myocardial strain and strain rate from speckle tracking provide load-independent measures<br>➔ E/e' ratio estimates left ventricular filling pressures<br>- Evaluation of pericardial fluid accumulation and its effects<br>- Assessment of catheter positions within vessels<br>- Evaluation of ductus arteriosus and foramen ovale shunts
Ord and Griksaitis [20], 2019	Fifteen-minute consultation: Using point of care ultrasound to assess children with respiratory failure	POCUS in pediatrics: Rapid evaluation of several differential diagnoses, using a predefined set of questions often leading to simple binary decisions; repeated scanning of the same patient to assess the impact of an intervention; improves patient safety during invasive procedures<br>- Benefits: No radiation, immediate results, minimal movement of the patient, safe and validated tool, quick feedback, relatively cheap, may reduce unnecessary interventions, promotes time at the bedside of the critically ill child, clear detailed pictures are relatively easy to obtain in the pediatric population

		- Drawbacks: accuracy altered by the skills of the practitioner, patient habitus alters accuracy, type of fluid is difficult to distinguish, majority of evidence base is in adult population, can be difficult to store images from government perspective, challenging to get training as a pediatrician<br>- POCUS sensitivity and specificity are superior or even considerable to chest x-ray in diagnosing pneumothorax, pleural effusion, consolidation, or pulmonary edema<br><br>Lung POCUS: linear probe in infants and a curved linear probe in older children<br>- Normal lung: bat-wing sign, pleural line, seahore sign, A-lines<br>- Pulmonary edema: normal pleural sliding, multiple B-lines (dense white lines radiating down from the pleura into the lung parenchyma); the sum of B-lines corresponds with the amount of edema<br>- Pulmonary consolidation: tissue-like sign (lung looks more like solid viscera, such as the liver), dynamic air bronchograms (air-filled echogenic alveoli due to dense consolidated surrounding lung), shred sign (the deeper border of consolidated lung tissue that makes contact with the aerated lung is shredded and irregular), lung pulse (is observed when the lung is not fully inflated, it is a measure of cardiac motion transmitted to the pleura through consolidated lung)<br>- Pneumothorax: absence of pleural sliding (motion M-Mode), no B-lines, barcode or stratosphere sign (M-Mode), presence of a "lung point" (visualization of the point that the visceral pleura starts to separate from the chest wall parietal pleura)<br>- Pleural effusion (Visualization of the fluid: appears black, between the lung parenchyma and the soft tissues), PLAPS point (lung edge floating within the dense black effusion), QUAD sign: shape drawn between the pleural line and the lung line (visceral pleura) at its deepest point, with the rib shadows either side)<br><br>Cardiac POCUS:<br>- Is the child well-filled? Inferior vena cava assessment (respiratory variation, no collapse), eyeball assessment of the filling status of the left ventricle<br>- Is the myocardial function normal? Visualization of the movement of the left ventricle<br>- Is there cardiac tamponade? Free fluid around the heart, right atrium collapses during diastole, right ventricle collapse on closure of outlet valves
Pan et al. [21], 2018	Neonatal and paediatric point-of-care ultrasound review	Airway POCUS: increased water content, decreased adipose tissue and muscle mass, lack of calcification of cartilaginous structures, overall smaller size and more superficial anatomy, resulting in better visualization of the laryngeal structures in children than in adults; trachea: anechoic (dark) round structure surrounded by hyperechoic (bright) cartilage rings

		- Vocal cord mobility: vocal cords appear as two hyperechoic linear structures that open and close with respiration and phonation; The false vocal cords are more prominent with a larger hyperechoic area due to their fat content. The true visual cords can be found more caudally.<br>- Endotracheal tube (ETT) positioning: 1) rule out esophageal intubation (endotracheal intubation: hyperechoic shadow within the hyperechoic cartilage of the trachea creating parallel hyperechoic arcs ('railroad sign'); esophageal intubation: hyperechoic circular structure adjacent to the trachea, often with a hyperechoic shadow within the lumen of the esophagus ('double trachea sign')), 2) rule our endobronchial intubation (scanning the bilateral anterior lungs for pleural sliding: unilateral pleural sliding (usually on the right side) indicates endobronchial intubation. Bilateral pleural sliding demonstrates an endotracheal intubation)<br>- ETT sizing: minimize laryngeal trauma and swelling; measure the transverse air-column diameter at the level of the cricoid cartilage to estimate the maximum size for the outer diameter of the ETT<br>Gastric POCUS: focuses on the antrum due to its more consistent and superficial localization and its lower proportion of air content<br>- Gastric content and volume: empty stomach (small, collapsed 'bull's eye', low risk for aspiration), thick fluid or solid contents (hyperechoic and homogenous like 'frosted glass', high risk for aspiration), gastric volumes equal to or less than 1.25 mL/kg in children are considered a low aspiration risk<br>- Placement of nasogastric and orogastric tube: the tip of the NGT/OGT should be visualized in the body or the antrum of the stomach; gastric tube is seen as a hyperechoic line or parallel hyperechoic lines<br>Lung POCUS: the anatomy in pediatric patients can produce higher quality images when visualizing deeper pulmonary parenchyma than in adults<br>- Pneumothorax: absence of pleural sliding, absence of a lung pulse, 'lung point' (interface between the pneumothorax space and the partially collapsed lung), absence of lung sliding due to pleural adhesions, apnea, or large lung bullae<br>- Pleural effusion: detect the amount of fluids as small as 5 mL, 'curtain sign', 'spine sign'<br>- Pediatric acute respiratory distress syndrome (PARDS): numerous B-lines in multiple lung sections bilaterally, disrupted and thickened pleura, consolidated lung tissue, and air bronchograms<br>- Transient tachypnoea of the newborn: numerous B-lines, consolidates lung tissue, areas of near-normal lung findings (e.g. A-lines)<br>- Meconium aspiration syndrome (MAS): presents like PARDS, but the lung ultrasound findings for MAS are dynamic due to changes in the spread of meconium plugs from mechanical ventilation, Serial images in multiple lung regions are required

		- Hemidiaphragmatic paralysis: visualizing diaphragmatic motion (using hepatic and splenic acoustic window) and thickening through M-mode scanning<br><u>Cardiac POCUS:</u> Focused assessed transthoracic echocardiography examination<br>- Identify cardiovascular causes of hypotension by checking for diminished systolic function, pericardial effusion, and valvular dysfunction<br>- assess fluid responsiveness in neonates with preserved left ventricular systolic function: measurement of variation on velocity-time integrals or of inferior vena cava collapsibility<br>- Assess pulmonary artery systolic pressure to evaluate for pulmonary hypertension<br>- Tricuspid regurgitation<br>- Persistent foramen ovale<br>- Inferior vena cava (IVC) examination: volume determination and responsiveness (IVC collapse during inspiration >50% in the setting of euvolemia and normal right atrial pressure; dilated IVC with decreased collapsibility (<50%) is a sign of increased right atrial pressure; A collapsed IVC may be suggestive of hypovolemia // IVC to descending aorta (Ao) ratio: IVC:Ao ratio = 1 euvolemia, IVC:Ao ratio < 0.8 intravascular depletion<br><u>Focused assessment with sonography for trauma (FAST) examination:</u> identifies the presence of free intraperitoneal and pericardial fluid for patients presenting with thoracoabdominal trauma<br><u>Abdominal POCUS:</u><br>- Necrotizing enterocolitis: free fluid, bowel wall thickness, pneumatosis intestinalis, portal venous gas, and vascular perfusion<br><br><u>!! Neonatal-specific ultrasound</u>:<br>- Rapid assessment of the neonate with sonography examination (RANS): to diagnose a decompensating neonate: pericardial effusion (anechoic fluid between the heart and the pericardial sac), pneumothorax and pleural effusion, central venous line malposition (linear, hyperechoic structure within the IVC lumen, correct position at the junction of the IVC and the right atrium), and severe intraventricular hemorrhage (hyperechoic mass within the ventricles)<br>- Endotracheal tube placement
Pawlowski et al. [59], 2023	Point-of-care ultrasound for non-vascular invasive procedures in critically ill neonates and children:	Essential bedside tool for real-time image guidance of invasive procedures in critically ill neonates and children<br>- Thoracentesis: POCUS shows a high diagnostic accuracy in identifying pleural effusions (type, size), and allows real-time visualization and guidance of needle insertion at the safest puncture site. Prevention of associated complications, such as iatrogenic pneumothorax and injuries to the visceral pleura, intercostal neurovascular bundle, and sub-diaphragmatic organs.

	current status and future perspectives	- Pericardiocentesis: POCUS shows a high diagnostic accuracy in identifying pericardial effusions (size, distribution around cardiac chambers, hemodynamic impact); subxiphoid, apical, and parasternal approaches for pericardiocentesis; visualizing the needle tip inside the pericardial sac and draining the effusion, and follow a drain insertion<br>- Paracentesis: Ultrasound can be used to confirm the presence of ascites and identify the optimal site for needle insertion; determine the depth of ascites and thickness of the abdominal wall, while ensuring the absence of underlying structures such as bowel, solid organs, or cysts; directly visualize needle insertion into the peritoneal cavity while avoiding surrounding structures<br>- Suprapubic aspiration: visualization of the bladder and real-time needle guidance of the procedure; assess bladder volume (10 mL is considered adequate) by measuring the length, width, and depth and multiplying by a correction coefficient<br>- Lumbar puncture: evaluation of infectious, neurologic, or metabolic diseases; Ultrasound identifies interspinous and subarachnoid spaces to guide needle placement and visualizes needle entry into the subarachnoid space between dura and arachnoid layers; ultrasound can identify patients with spinal cord abnormalities and those who may have a low probability of a successful tap<br>- Endotracheal tube placement: visualize the trachea and esophagus: An empty esophagus and widened subglottis are consistent with tracheal intubation; ETT can be visualized in the esophagus in the event of esophageal intubation; Observation of bilateral pleural sliding on subsequent lung ultrasound suggests appropriate positioning above the carina.<br>- Nasogastric tube placement: Gastric POCUS can be used to confirm the correct location of nasogastric tube tip, which can be directly visualized as a double hyperechoic track into the gastric antrum
Pérez-Calatayud and Carillo-Esper [53], 2023	Role of gastric ultrasound to guide enteral nutrition in the critically ill	Early initiation of enteral nutrition (EN) is often not feasible due to the high prevalence of gastrointestinal dysfunction in the critically ill. EN is essential for patients in the ICU, and is considered a standard of care. The benefits of enteral nutrition include preserving the gastrointestinal mucosa's structure and function, decreasing catabolic response to injury, and reducing risk of bacterial translocation. Gastric POCUS could become a tool for the management and monitoring of enteral nutrition in critically ill patients. Gastrointestinal dynamics provide a valuable guide to initiate enteral nutrition, predict feeding intolerance, and aid in following treatment response.<br>- Assessment of gastric content and volume (evaluation of aspiration risk assessment): 'risk stomach' gastric volume > 1.5 ml/kg of body weight, measured at the level of the gastric antrum.<br>- Ultrasound meal accommodation test: identify patients who will develop feeding intolerance by monitoring the gastric response to meal stimulation<br>- Evaluate gastrointestinal integrity: gastrointestinal diameter, mucosal thickness, peristalsis, and blood flow

		- Guide enteral nutrition: accurate confirmation of the feeding tube position, monitoring the gastric residual volume, assessing gastrointestinal motility, and monitoring the nutritional status of patients.
Potter and Griksaitis [39], 2019	The role of point-of-care ultrasound in pediatric acute respiratory distress syndrome: emerging evidence for its use	Pediatric acute respiratory distress syndrome (PARDS):<br>- The 2015 PALICC definition of PARDS requires chest imaging (CXR, CT) to diagnose the presence of new pulmonary infiltrate(s) consistent with acute pulmonary parenchymal disease<br>- benefits of POCUS: lack of ionizing radiation, immediate feedback, promoting time at the bedside of the critically unwell child, and ease of serial assessments<br>- POCUS can support the diagnosis of pediatric ARDS, assess for complications, monitor progression and guide management<br>- Mortality of PARDS is as high as 40% in pediatric patients (complications: pneumothorax, ventilator associated pneumonia, ventilator-induced lung injury)<br>- <u>Normal lung POCUS features:</u><br>➔ Bat-wing sign (upper and lower adjacent ribs combined with pleural line)<br>➔ Pleural line is the horizontal dense, crisp sharp white line; should be seen shimmering and sliding back and forth to indicate normal movement of the pleura; interface between pleural surfaces<br>➔ Seashore sign (M-mode): Pleural sliding<br>➔ A-lines are horizontal reverberation artifacts arising from pleural line, indicating clear lungs when multiple and parallel.<br>- <u>PARDS-relevant abnormal lung POCUS features:</u><br>➔ Interstitial (alveolar) edema: B-Lines (comet tail artifacts (dense, white, vertical lines) arising from pleural line to screen, obliterating the A-lines; they can represent fluid in the alveoli or interstitial thickening; can be seen in pulmonary edema, ARDS, fibrosis and transient tachypnoea of the newborn)<br>➔ Consolidation and lung parenchymal disease (dynamic air bronchograms; tissue-like sign (appearance like solid viscera); shred-sign (broder of consolidated and aerated lung); lung pulse (movement of pleura in relation to transmissions of cardiac pulsations), absent pleural sliding (barcode-sign))<br>! Consolidation can be seen predominantly posterior (dependent regions); anterior B-lines of differing intensity and subpleural consolidation can be seen; normal lung present (‘spared areas’) in the non-dependent regions<br>! Sign for pneumonia: in pediatric population the most common cause of PARDS is pneumonia<br>➔ Pneumothorax (absent pleural sliding (barcode-sign), absence of B-lines, no lung-pulse, lung point (point at which the visceral and parietal pleural start to separate))

Rath et al. [22], 2023	Point-of-Care Ultrasound in Neonatology in India: The Way Forward	Utility as a diagnostic tool: rapid assessment in a crashing neonate<br>- Cardiac POCUS: preload assessment, fluid responsiveness, cardiac function assessment, pericardial effusion, patent ductus atreriosus assessment and treatment monitoring, pulmonary hypertension assessment and treatment monitoring, recognition of abnormal cardiac anatomy<br>- Cranial POCUS: estimation of cerebral blood flow velocities (resistive and pulsatility indices), parenchymal/intraventricular hemorrhage, cerebral midline shift, hydrocephalus<br>- Lung POCUS: respiratory distress syndrome, transient tachypnea of the newborn, predict surfactant need, pneumonia, air leak syndromes, pleural effusion, lung edema, pneumothorax<br>- Abdominal POCUS: necrotizing enterocolitis (bowel viability assessment: peristalsis, vascular perfusion, pneumatosis intestinalis, portal venous gas, bowel-wall-thickness, free fluid), bladder assessment for anuria or urinary retention<br>Utility as a tool to aid in procedures: improves success and decreases complications associated with the procedures, reduction of malpositioning, real-time guidance<br>- Central line tip placement and localization: umbilical central lines, peripherally inserted central lines<br>- Endotracheal tube localization<br>- Lumbar puncture<br>- Suprapubic tap: bladder visualization for suprapubic urine collection for cultures<br>- Pericardiocentesis and thoracocentesis: neonates with hydrops or congenital pleural effusion<br>POCUS in crashing neonates: in the following critical situations, POCUS would play a useful and critical role:<br>- Infants unresponsive in a neonatal resuscitation protocol<br>- Unexplained acute respiratory failure or worsening hypoxemia unresponsive to usual respiratory support<br>- Unexplained acute circulatory shock or worsening hypotension, lactic acidosis, oliguria, unresponsive to volume expansion, and vasopressors<br>- Unexplained drop in hemoglobin >20% in 24 hours with suspicion of acute bleeding
Sehgal and Menahem [34], 2023	The left ventricle in well newborns versus those with perinatal asphyxia, haemodynamically significant ductus arteriosus or fetal growth restriction	POCUS helps differentiate normal transition of fetal to neonatal circulation and that resulting from neonatal disorders like perinatal asphyxia, hemodynamically significant patent ductus arteriosus, and uteroplacental insufficiency which can adversely affect left ventricular (LV) function.<br>- Characterization of the shift from RV to LV dominance by POCUS<br>- perinatal asphyxia: evaluation of LV size, contractility, shortening fraction, and output can help detect the reduced cardiac function<br>- Patent ductus arteriosus: assessment of ductal shunting and determining the need for ligation<br>- Uteroplacental insufficiency/fetal growth restricted infants: higher systolic pressures, hypertrophied heart, and increased arterial stiffness

Shepherd et al. [36], 2022	Neonatal POCUS: Embracing our modern day "stethoscope"	Ultrasound may improve diagnostic accuracy and imaging acquisition time, result in medical innovation, and improve overall outcomes. POCUS has become a natural part of day-to-day and has become as essential as the stethoscope.<br>- Cardiac POCUS: central line position, detection and guidance for drainage of pericardial effusion, qualitative assessment of cardiac filling and function, and evaluation for patency of ductus arteriosus<br>- Lung POCUS: A-lines (hyperechoic horizontal lines which signify air within the lung, absent in pneumothorax), B-lines (hyperechoic vertical lines that arise from the pleural line, represent interstitial lung disease (fluid within the lung, atelectasis)), Lung sliding (twinkling effect as the parietal and visceral pleura move past each other, absent in pneumothorax and pleural effusion), lung point (M-mode, where the pleura separate as in pneumothorax, normal "seashore sign" turns into the "stratosphere sign"), double lung point (the point at which very compact B-lines transition to less compact B-lines, seen in transient tachypnea of the newborn), pleural line (abnormality in pneumonia)<br>- Abdominal POCUS: free fluid (hemorrhage, ascites), necrotizing enterocolitis (focal fluid collections, complex ascites, absent peristalsis, pneumoperitoneum, bowel wall echogenicity, bowel wall thinning, absent perfusion, bowel wall thickening, and dilated bowel)<br>- Cranial POCUS (coronal and sagittal views performed through the anterior fontanelle, and axial views to interrogate the posterior fossa): hemorrhage<br>- Bladder POCUS: urinary retention, bladder volume, estimate oliguria and anuria, suprapubic aspiration<br>- Central catheter placement (Umbilical catheters and peripherally inserted central catheters for intravenous medication and nutrition)<br>- Endotracheal tube position<br>- Lumbar puncture (provide anatomic confirmation helpful for both marking insertion site and for direct visualization for needle insertion)
Singh et al. [23], 2021	The evolution of cardiac point of care ultrasound for the neonatologist	Cardiac POCUS provides real-time physiological and hemodynamic information at the bedside to aid clinical decision-making for neonatal emergencies. It allows rapid goal-directed cardiac assessment that can help guide resuscitation and management. International guidelines have defined the scope and training standards for more advanced neonatal echocardiography (NPE/TNE), but not for basic cardiac POCUS. There is a need to establish standardized training curricula and certification pathways specifically for neonatal cardiac POCUS.<br>- Qualitative assessment of global cardiac systolic function and volume status<br>- Guiding resuscitation efforts by evaluating cardiac contractility, heart rate, and cardiac output<br>- Evaluation of patent ductus arteriosus, including shunt direction and size<br>- Assessment of tricuspid and mitral regurgitation<br>- Detection of pericardial effusions and cardiac tamponade, guiding of pericardiocentesis

		- Guiding placement and assess position of umbilical and peripherally inserted central catheters<br>- Diagnosis of congenital heart disease such as ventricular septal defects
Stewart et al. [2], 2022	Use of Point-of-Care Ultrasonography in the NICU for Diagnostic and Procedural Purposes	POCUS could be considered an extension of the physical examination performed at the bedside by non-radiologists to assist procedures and perform a time-sensitive assessment of the symptomatic patient with immediate identification of pathologic processes that can guide resuscitative and lifesaving interventions.<br>Advantages of POCUS: no ionizing radiation, readily available, no sedation, less expensive than MRI/CT, portability, dynamic<br><u>Diagnostic applications of POCUS:</u><br>- Emergency:<br>➔ Cardiac: non-response to Neonatal Resuscitation Program protocol without an identifiable reason, acute circulatory shock (hypotension, lactate acidosis, oliguria) unresponsive to initial resuscitation maneuvers (volume expansion, cardiovascular medication)<br>➔ Lung: acute respiratory distress syndrome, worsening hypoxemia unresponsive to usual respiratory support<br>➔ Unexplained drop in hemoglobin >20% in 24 h with suspicion of acute bleeding (intracranial hemorrhage, hemopericardium, hemothorax, subcapsular, abdominal, or splenic hemorrhages)<br>- Lung POCUS: based on air-fluid interface-related artifacts: A-lines (reflect aeration); B-lines (reflect thickened septa by interstitial or alveolar fluid); air bronchograms (in atelectasis and in consolidation, when associated with hypoechoic areas and pleural irregularities)<br>➔ Respiratory distress syndrome (RDS)<br>➔ Transient tachypnea of the newborn (TTN)<br>➔ Guiding the need for surfactant administration in preterm infants<br>➔ Pneumothorax: (1) absence of sliding sign of the pleural line; (2) complete absence of B lines (ie, only Alines); (3) presence of a lung point and (4) presence of a barcode sign in M-mode imaging<br>➔ Lung consolidation: the presence of a nonaerated area or lung parenchymal portion filled with fluid ('hepatic' appearance, abnormal pleural line, bronchograms); caused by atelectasis, inflammatory processes (pneumonia), pulmonary edema, or acute pulmonary hemorrhage<br>➔ Pleural effusion: anechoic (black) space between the parietal and visceral pleura; evaluating the characteristics of the fluid (simple anechoic, granular, fibrinous, septated, loculated, etc) and diagnosing the nature of the effusion possible by POCUS<br>➔ Congenital diaphragmatic hernia (CDH): absence of the diaphragmatic echogenic line, absence of A-lines, absence of the pleural line and lung sliding on the side of the CDH, presence of bowel loops with peristalsis, and possible detection of liver or spleen in the hemithorax

		➔ Diaphragmatic motion assessment: monitoring the diaphragmatic movement via the US probe placed subcostally at the anterior axillary line for the right hemidiaphragm and subcostally at the posterior axillary line for left hemidiaphragm. M-mode can be used for quantitative assessment of excursion with 0.5 to 1 cm as acceptable range in neonates.<br>- Cardiac POCUS: time-sensitive assessment of the symptomatic patient and immediate identification of pathologic processes that can be diagnosed rapidly with standard basic echocardiographic views<br>➔ Cardiac tamponade and pericardial effusion + guiding of pericardiocentesis<br>➔ Assessment of the position of the umbilical venous catheter and peripherally inserted central catheters: evaluation of central line-related complications (ie, pericardial effusions, air embolism, thrombus at the tip of the catheter), real-time adjustment of the tip position<br>➔ Assessment of global cardiac systolic function/contractility<br>➔ Assessment of cardiac filling and fluid status: through "eyeballing" (qualitative assessment) an underfilled heart (decreased preload) or volume overload (increased preload); hemorrhage, dehydration, third space losses or severe pulmonary hypertension can lead to low ventricular volume status (at the end of systole when the ventricular walls are touching each other, and the ventricular cavity is collapsing)<br>➔ Assessment of pulmonary hypertension: ventricular asymmetry with dilated right ventricle compared with the left ventricle, paradoxical movement and shape of the interventricular septum at the end of systole<br>- Abdominal POCUS:<br>➔ Assessment of abdominal bleeding or ascites: localizing intraabdominal fluid; safe guiding of paracentesis<br>➔ Necrotizing enterocolitis: pneumatosis intestinalis, portal venous gas, bowel wall thickness, bowel wall signature, peritoneal ascitic fluid, peristalsis, hyperemia followed by ischemia, dilated loops, pneumoperitoneum<br>- Cranial POCUS:<br>➔ Intraventricular hemorrhage<br>➔ Ischemia (cerebral blood flow)<br>➔ Monitoring of intracranial pressure through anterior fontanelle<br><u>Procedural applications of POCUS:</u><br>- Vascular access: peripherally inserted central catheter, umbilical central venous/arterial catheters, peripheral arterial/venous lines → adequate catheter size by measuring the vessel, following line insertion to evaluate and adjust tip position in real-time, reduce risk of thrombosis<br>- Lumbar puncture: identifying landmarks before the procedure (i.e. conus medullaris), depth required to reach cerebrospinal fluid, level where the spinal canal begins to narrow, or as the needle is introduced and

		advanced into the spinal canal → higher success rates, less traumatic, shorter time to a successful LP, and lower patient pain scores<br>- Fluid drainage: paracentesis (confirmation of ascites, identification of the largest ascitic pocket, localization of epigastric vessels to avoid undesired punctures of adjacent structures and bleeding), pericardiocentesis, thoracentesis (chest tube placement guidance or needle aspiration in neonates with pleural effusion and pneumothorax), and suprapubic aspiration (size and location of the bladder as well and volume of urine)<br>- Endotracheal tube placement
Su et al. [30]	Death and Ultrasound Evidence of the Akinetic Heart in Pediatric Cardiac Arrest	Assessment of organ injury in cardiac arrest (CA) and the determination of cardiac criteria for death<br>- Causes of CA: massive pulmonary embolism, pericardial tamponade<br>- Cardiac criteria of death: cardiac akinesis (absence of contractility) with loss of anterograde flow (>2 min)
Subramaniam et al. [46], 2019	Identifying infant hydrocephalus in the emergency department with transfontanellar POCUS	Hydrocephalus carries significant morbidity in the infant population. Although clinical symptoms are often nonspecific, hydrocephalus is easily identified using transfontanellar sonography.<br>- Visualization of the ventricular system with transducer frequencies ranging between 5 MHz to 10 MHz.<br>- Open anterior fontanelle (up to 12-18 months) acts as an acoustic window<br>- Dilated ventricular system<br>➔ Widening or 'ballooning' of the anterior horn of the lateral ventricles, rounding or 'Mickey Mouse ears' appearance (>10mm)<br>➔ Widening of the temporal horn of the lateral ventricles (>3mm)<br>➔ 3rd ventricle in sagittal plane (>1mm)<br>➔ Bifrontal index >0.5 (ratio of the widest measurement of anterior horns to the widest measurement of the inner table of the skull)
Tsou et al. [52], 2019	Accuracy of point-of-care ultrasound and radiology-performed ultrasound for intussusception: A systematic review and meta-analysis	Ultrasonography for intussusception has a sensitivity: 0.98 (95% CI: 0.96–0.98) and a specificity: 0.98.<br>Current evidence suggests POCUS has a high diagnostic accuracy for intussusception not significantly different from that of radiology-performed ultrasound (RADUS).<br>- Intussusception appears on ultrasound as a target or doughnut-like mass consisting of layers of bowel sliding in on itself.<br>- The intussusceptum (inner layer of the bowel) can be seen within the intussuscipiens (outer layer of the bowel acting as a sheath).<br>- On transverse view there is typically a concentric arrangement of alternating echogenic (bowel wall) and anechoic (bowel lumen) rings.<br>- On longitudinal view, the "pseudokidney" sign is seen as two parallel echogenic lines representing the intussusceptum wall and outer intussuscipiens wall, separated by an anechoic space.

		- Associated findings: bowel wall thickening, peripheral fat stranding, and fluid-filled bowel proximal to the intussusception.<br>- Doppler may show alternating areas of increased and decreased vascularity within the intussusception layers.
Valla et al. [54], 2022	Gastric Point-of-Care Ultrasound in Acutely and Critically Ill Children (POCUS-ped): A Scoping Review	- Assess gastric emptying and gastric volume/contents<br>➔ Gastric antrum cross-sectional area (CSA) of 219 mm² in the supine position and 307 mm² in the right lateral decubitus position allows discrimination between an empty or full stomach<br>➔ IImpact of different feed types (breast milk, fortifiers, and thickeners) on gastric emptying: mean gastric emptying time of breast milk <4 h, after formula feeding 93min, <1 h after clear fluids<br>➔ Risk for pulmonary aspiration prior to sedation or anesthesia or during surgery<br>- Gastric foreign body ingestion<br>- Nasogasatric tube placement<br>- Hypertrophic pyloric stenosis diagnosis<br>- Gastric insufflation during mechanical ventilatory support
