# Supplementary material for: Applications of Point-of-Care-Ultrasound in Neonatology: A Systematic Review of the Literature
Source: Life (Basel). 2024 May 22;14(6):658. doi: 10.3390/life14060658 (PMC11204601; doi:10.3390/life14060658)
Supplement: Supplementary file 1 [file life-14-00658-s001.zip › Supplementary Table S3.pdf]

Table S3. Guidelines considering Point-of-care ultrasound (POCUS) in neonatology

Author/year/title		Recommendation of applying POCUS in neonatology
Elsayed et al. [31], 2023	Point-of-care ultrasound (POCUS) protocol for systematic assessment of the crashing neonate—expert consensus statement of the international crashing neonate working group	- Crashing Neonate Protocol (CNP)<br>➔ CNP is specifically designed for use in neonatal emergencies leading to significant cardiorespiratory instability and can be used in both term and pre-term neonates who are either "crashed" (needing resuscitation) or "crashing" (likely to need resuscitation if not stabilized soon) infants.<br>➔ adjunct to the current recommendations for neonatal resuscitation, with pre-defined steps.<br>➔ CNP proposes a stepwise systematic targeted assessment, focused on gaining information regarding the underlying pathophysiology in unexplained "crashing" neonates, with simple basic ultrasound views that are easily reproducible and can be learned with focused training<br>(a) Lung-POCUS assessment of pulmonary emergencies (pneumothorax, pleural effusion, or lung atelectasis)<br>(b) Cardiac-POCUS assessment of shock and hemodynamic instability<br>(c) Cranial-POCUS assessment for acute brain hemorrhage<br>(d) Abdominal-POCUS assessment of peritoneal or subcapsular bleeding, gut injury, or bowel ischemia<br>(e) Assessment of central line-related complications by Central-Line POCUS
Hardwick and Griksaitis [15], 2021	Fifteen--minute consultation: Point of care ultrasound in the management of pediatric shock	- POCUS uses a predefined sequence of focused scans. This allows the trained clinician to better differentiate between the causes of pediatric shock in a timely manner, and subsequent reassessment can provide information about the patients response to treatment.<br>- The pathophysiology of shock can be summarized as an acute state of energy failure, which prevents the metabolic demands of cells from being met.<br>- In the pediatric patient, cardiac output (CO) is more dependent on heart rate (HR) than on stroke volume (SV) due to the lack of ventricular muscle mass and decreased ventricular elastance. Consequently, tachycardia is the child's principal means of compensating to maintain an adequate CO. BP can often be preserved, so BP is often a poor indicator of cardiovascular homeostasis.<br>- Key questions to answer with POCUS when dealing with a shocked child<br><br>Is there a preload problem?<br>► Is there evidence of pulmonary edema?<br>► Is the heart full or empty? Is the IVC distended or collapsible?<br>► Are there signs of fluid loss (e.g. effusions, bleeds)?<br>Is there a contractility problem?<br>► Are the ventricles ejecting blood adequately?<br>► Are the atria and/or ventricles dilated?

		Is there an obstructive problem?<br>► Cardiac tamponade?<br>► Tension pneumothorax?<br>► Pleural effusions?<br><br>Pediatric shock POCUS protocol<br>(a) Lung POCUS: B-lines (pulmonary edema)? Pneumothorax? Pleural effusions? Consolidation (Source of sepsis)?<br>(b) Cardiac POCUS: Ventricles well filled? Cardiac function adequate? Elevated pulmonary pressure? Evidence of pericardial effusion? Evidence of cardiac tamponade?<br>(c) Inferior vena cava POCUS: Distended? Collapsibility? Respiratory variation in size?<br>(d) Abdominal POCUS (four quadrant assessment): free intra-abdominal fluid? Evidence of urine production? Evidence of renal infection (Source of sepsis)? Hepatic hemangioma?<br>(e) Cranial POCUS (via anterior fontanelle): Subdural bleeds? Intra-cranial bleeds? Hydrocephalus? Vein of Galen?
Maddaloni et al. [17], 2023	The role of point-of-care ultrasound in the management of neonates with congenital diaphragmatic hernia	Management of neonates with a congenital diaphragmatic hernia (CDH):<br>- The use of POCUS should be encouraged to improve ventilation strategies, systemic perfusion, and enteral feeding, and to intercept any early signs related to future neurodevelopmental impairment.<br>- CDH-POCUS protocol: serial evaluation of four functional areas<br>(a) Cardiac POCUS: left ventricle function, right ventricle function, pulmonary hypertension<br>(b) Lung POCUS: lung volumes, post-operative pleural effusion or pneumothorax, endotracheal tube placement, diaphragm evaluation<br>(c) Splanchnic POCUS: mesenteric blood flow, signs of malrotation or volvulus, signs of necrotizing enterocolitis, vena cava thrombosis assessment, kidneys position, renal blood flow<br>(d) Cranial POCUS: cerebral blood flow, intracranial bleeding, severe brain lesions<br>➔ Three standardized timelines for CDH-POCUS evaluations: 1) the first day of life (immediately following postnatal stabilization), 2) the time leading up to surgery, and 3) after surgical correction
Singh et al. [24], 2020	International evidence-based guidelines on Point of Care Ultrasound (POCUS) for critically ill neonates and children issued by the POCUS Working Group of	An international panel developed 41 recommendations for cardiac, lung, vascular, cerebral, and abdominal POCUS based on a literature review. Most recommendations (28/41) were based on moderate quality evidence (Grade B-C). Guidelines provide evidence on the optimal use of POCUS to optimize clinical care, training, and research. It encompasses for instance:<br>- Cardiac POCUS: hypotension, shock, arrhythmias, and structural defects<br>- Lung POCUS: respiratory distress, pneumonia, pleural effusions

	the European Society of Paediatric and Neonatal Intensive Care (ESPNIC)	- Vascular POCUS: DVT, line placement<br>- Cerebral POCUS: head trauma, intraventricular hemorrhage<br>- Abdominal POCUS: bowel perforation, ascites, abscess drainage
Yousef et al. [25], 2022	"Playing it SAFE in the NICU" SAFE-R: a targeted diagnostic ultrasound protocol for the suddenly decompensating infant in the NICU	SAFE-R: targeted ultrasound protocol specifically designed for the suddenly decompensating infant in the NICU for rapid screening of the most common life-threatening complications needing immediate attention.<br>(a) Cardiac POCUS:<br>- myocardial dysfunction? cardiac contractility is rapidly evaluated using an "eyeball" method → echocardiogram<br>- cardiac tamponade? → needle aspiration<br>(b) Lung POCUS:<br>- tension pneumothorax? No lung sliding, only A-Lines, no B-Lines, Lung point → needle aspiration<br>- pleural effusion? → needle aspiration<br>(c) Abdominal POCUS:<br>- acute critical aortic occlusion (coarctation or thrombosis)? No pulsatile abdominal aorta → expert needed<br>- acute abdominal complications (intestinal perforation, hemoperitoneum)? Abdominal free fluid → targeted workup<br>(d) Cranial POCUS:<br>- intraventricular hemorrhage? → supportive management
